# Neurofilament Light Chain (NfL) in Neurodegenerative Diseases: Biological and Clinical Significance, Multi-Omics Integration, and AI-Driven Biomarker Modeling for Precision Therapy

**DOI:** 10.3390/ph19091326

**Published:** 2026-08-22

**Authors:** Nawaf Alshammari, Reyaz Hassan, Mitesh Patel, Mohd Adnan

**Affiliations:** 1 Department of Biology, College of Science, University of Ha’il, Ha’il P.O. Box 2440, Saudi Arabia; naib.alshammari@uoh.edu.sa; 2 King Salman Center for Disability Research, Riyadh 11614, Saudi Arabia; 3 Pharmaceutical Chemistry Division, Department of Pharmaceutical Sciences, University of Kashmir, Hazratbal, Srinagar 190006, Jammu and Kashmir, India; reyazhassan.scholar@kashmiruniversity.net; 4 Department of Bioinformatics, Faculty of Engineering and Technology, Marwadi University, Rajkot 360003, Gujarat, India

**Keywords:** neurofilament light chain, neurodegenerative diseases, disability, neuroaxonal injury, multi-omics integration, artificial intelligence, multimodal biomarkers, precision therapy

## Abstract

Neurodegenerative diseases represent a major cause of disability and death, but early diagnosis, prognosis, and therapeutic monitoring are challenging due to biological heterogeneity and the absence of disease-specific biomarkers. Neurofilament light chain (NfL) is a highly sensitive fluid biomarker of neuroaxonal injury with well-established clinical utility in selected neurological disorders, especially in disease monitoring and prognostic evaluation. However, since NfL is not disease-specific, the interpretation has to be integrated with complementary molecular, imaging, and clinical biomarkers. Recent advances in genomics, epigenomics, transcriptomics, proteomics, metabolomics, microbiome profiling, and neuroimaging provide complementary information about the molecular and biological processes underlying neurodegeneration. Artificial intelligence (AI) and machine-learning approaches also allow the integration of these heterogeneous datasets for multimodal biomarker modeling. This review examines the biological and clinical relevance of NfL across major neurodegenerative diseases and critically discusses its combination with multi-omics, neuroimaging, and AI-based approaches. Special emphasis is placed on disease monitoring, prognosis, patient stratification, and therapeutic-response modeling, distinguishing established clinical applications from emerging research directions. The present review also addresses ongoing methodological challenges, including assay standardization, data harmonization, model interpretability, multicenter validation, and clinical translation. Finally, future potential is discussed for NfL-based multimodal biomarker frameworks in precision neurology.

## 1. Introduction

Neurodegenerative diseases (NDs) are leading causes of disability and death worldwide, such as Alzheimer’s disease (AD), Parkinson’s disease (PD), amyotrophic lateral sclerosis (ALS), multiple sclerosis (MS), frontotemporal dementia (FTD), and Huntington’s disease (HD) [1]. Despite their different clinical manifestations, these disorders share a number of common pathological features such as protein aggregation, neuroinflammation, mitochondrial dysfunction, oxidative stress, and progressive neuroaxonal degeneration [2,3]. Since irreversible neuronal damage often develops after years of clinical symptoms, the identification of reliable biomarkers for early diagnosis, disease monitoring, and therapeutic evaluation is still a major unmet clinical need [4].

Neurofilament light chain (NfL) is a structural component of the neuronal cytoskeleton and is one of the most important fluid biomarker of neuroaxonal injury. NfL is released into the cerebrospinal fluid and then into the bloodstream following neuronal injury and can be quantified by ultrasensitive assays such as single-molecule array (Simoa) [5,6]. Increased blood and CSF NfL levels have been strongly associated with disease severity, progression, and treatment response in a number of neurodegenerative diseases [7,8]. However, as NfL is a marker of neuroaxonal damage and not disease-specific molecular mechanisms, its utility as a stand-alone biomarker is inherently limited [9].

Recent advances in multi-omics technologies, such as genomics, epigenomics, transcriptomics, proteomics, metabolomics, and microbiome profiling, have provided complementary insights into the molecular complexity of neurodegeneration [10,11]. The integration of these approaches with NfL may offer a wider biological context for the interpretation of neuroaxonal injury and disease heterogeneity [10,12]. The growing availability of large-scale multi-modal data has also accelerated the use of artificial intelligence (AI) in neurodegenerative disease research. Machine learning and deep learning algorithms can combine heterogeneous biological, imaging, and clinical data, identify complex biomarker interactions, and produce predictive models for diagnosis, prognosis, and patient classification [13]. The combination of NfL with multi-omics and AI-driven neuroimaging data is emerging as a promising approach to advance precision neurology through biologically informed and personalized disease profiling [12,14].

Hence, this review highlights the biological and clinical significance of NfL in major neurodegenerative diseases and discusses its integration with multi-omics technologies and AI-based analytical approaches. The review also outlines current clinical applications, existing challenges, and future directions and proposes an NfL-centered precision neurology framework to enable comprehensive neurodegeneration profiling, improved risk prediction, and personalized therapeutic decision-making.

## 2. Methodology

This review was carried out as a narrative review to review and critically discuss the current evidence on the role of NfL in neurodegenerative diseases, with a special focus on its integration with multi-omics technologies and AI-based analytical approaches. A comprehensive literature search was carried out in PubMed/MEDLINE, Scopus, Web of Science, Embase, and Google Scholar to find relevant studies. The search was mainly limited to articles published from January 2015 to January 2025. However, several landmark studies published prior to this period were also included where they provided important information on the biology of NfL, assay development, or established the clinical significance of NfL as a biomarker. Searches were performed using combinations of the keywords “neurofilament light chain”, “NfL”, “neurodegenerative diseases”, “Alzheimer’s disease”, “Parkinson’s disease”, “amyotrophic lateral sclerosis”, “multiple sclerosis”, “frontotemporal dementia”, “Huntington’s disease”, “multi-omics”, “genomics”, “transcriptomics”, “proteomics”, “metabolomics”, “microbiome”, “neuroimaging”, “artificial intelligence”, “machine learning”, “deep learning”, “precision medicine” and “biomarker”. The search terms were combined with Boolean operators (AND and OR) to improve retrieval of relevant publications.

Only original research articles, clinical studies, systematic reviews, meta-analyses, and high-quality review articles that are peer-reviewed and relevant to the scope of this review were considered. Conference abstracts, editorials, commentaries, duplicate publications, non-English articles, and studies that did not focus on NfL or its relationship with neurodegenerative diseases, multi-omics, or AI-based approaches were excluded. The retrieved articles were screened by their titles, abstracts, and full texts to assess their relevance. More focus was given to recent studies, large clinical trials, multicenter studies, and publications that have made significant contributions to the understanding of the biological and clinical relevance of NfL, improvements in multi-omics technologies, and AI-driven biomarker modeling. The selected literature was then critically reviewed and organized into thematic sections that addressed NfL biology, clinical applications, multi-omics integration, AI-based analytical strategies, current challenges, and future perspectives in precision neurology.

## 3. NfL Biology and Clinical Significance

NfL is one of the most widely investigated biomarker of neuroaxonal injury and neurodegeneration. Due to its neuronal specificity, high abundance in axons, and its measurable release into biological fluids after neuronal damage, NfL has gained great importance in both research and clinical settings [5,8,15]. Recent advances in technology have enabled the detection of NfL in cerebrospinal fluid (CSF) and blood with high sensitivity, thereby allowing its use as a minimally invasive biomarker for diagnosis, disease monitoring, and therapeutic assessment in a wide range of neurological disorders [16]. To interpret the clinical relevance of NfL in neurodegenerative diseases, it is essential to understand its biological properties and the mechanisms controlling its release, as well as the methods to quantify it [17].

### 3.1. Structure and Function of the Neurofilaments

Neurofilaments are key intermediate filament proteins and an important part of the neuronal cytoskeleton. They are predominantly localized in axons where they provide structural support, maintain axonal caliber, and facilitate intracellular transport. Neurofilaments (NFs) are members of the type IV intermediate filaments (IFs) and consist of three major subunits, neurofilament light chain (NfL), neurofilament medium chain (NfM), and neurofilament heavy chain (NfH). Additional associated proteins such as α-internexin in the central nervous system and peripherin in the peripheral nervous system further neurofilament assembly and functional diversity [17,18].

Among these subunits, NfL is the primary structural scaffold necessary for neurofilament assembly. The NfL protein is made up of a conserved α-helical rod domain flanked by amino-terminal head and carboxy-terminal tail domains. The assembly of neurofilaments proceeds through a highly regulated process with NfL monomers first assembling into coiled-coil dimers, which then assemble to form tetramers, protofilaments, and mature neurofilament structures. These filamentous networks provide mechanical stability to neurons and are involved in maintaining axonal architecture [17,18].

**Figure 1:**
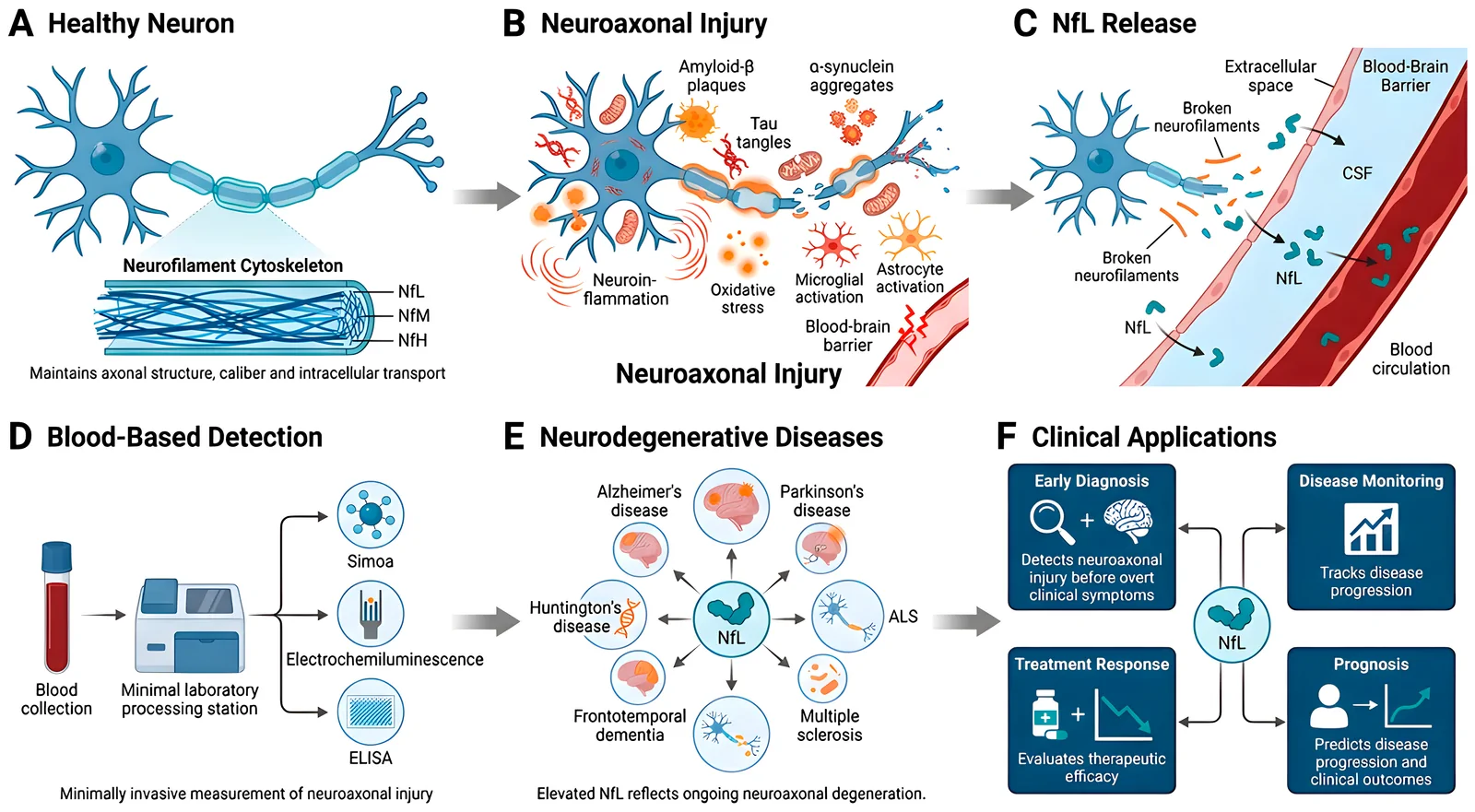
Neurofilament light chain (NfL) as a biomarker of neuroaxonal injury and its clinical relevance in neurodegenerative diseases. (**A**). Neurofilaments (NfL, NfM, and NfH) maintain axonal structure and function in healthy neurons. (B). Neuroaxonal injury associated with protein aggregation, oxidative stress, neuroinflammation, and blood–brain barrier dysfunction is associated with neurofilament breakdown. (**C**). Released NfL enters the cerebrospinal fluid (CSF) and bloodstream following neuronal damage. (**D**). Blood NfL can be measured using sensitive assays such as Simoa, electrochemiluminescence, and ELISA. (**E**). Elevated NfL levels are observed across multiple neurodegenerative diseases. (**F**). Clinically, NfL supports early diagnosis, disease monitoring, treatment response assessment, and prognosis.

In addition to their structural role, neurofilaments are involved in a variety of important neuronal functions including axonal transport, conduction velocity, and synaptic integrity [18]. The sensitivity of NfL as a marker of neuroaxonal injury is due to the abundance of neurofilaments in large myelinated axons. Under physiological conditions, only low amounts of NfL are released into extracellular fluids because of normal neuronal turnover. However, NfL release into CSF and blood circulation can be dramatically increased by the pathological processes associated with neurodegeneration [5,8,17] (Figure 1A–F).

### 3.2. Mechanism of NfL Release During Neurodegeneration

Elevated NfL levels in CSF and blood are primarily a marker of active neuroaxonal injury. NfL is not disease-specific, but its release is closely linked to pathological processes that lead to neuronal injury and degeneration. The release of NfL from damaged neurons has been linked to several interconnected mechanisms [5,8,19].

#### 3.2.1. Axon Damage

Axonal injury is considered the primary source of NfL release in neurological diseases and is strongly associated with increased NfL concentrations. Neurodegenerative diseases are associated with progressive disruption of axonal cytoskeletal integrity, which may result from protein aggregation, reduced axonal transport, mitochondrial dysfunction, and metabolic stress [20]. Neurofilament networks become structurally destabilized, which contributes to axon fragmentation and leakage of NfL into the extracellular environment [5,7,19]. Often, the degree of axonal degeneration correlates with the extent of NfL increase, making it a useful biomarker of disease severity and progression. In diseases characterized by prominent axonal degeneration, such as ALS and multiple sclerosis, NfL concentrations can rise markedly and correlate strongly with clinical outcomes. Similar trends have been observed in AD, FTD, and HD, supporting the utility of NfL as a pan-neurodegenerative marker of neuronal injury [8,15].

#### 3.2.2. Synaptic Dysfunction

Many reported studies support that synaptic dysfunction is an early pathological feature in numerous neurodegenerative diseases. Changes in synaptic connectivity, neurotransmission, and neuronal communication may precede overt neuronal loss by years. Progressive synaptic dysfunction is associated with cytoskeletal instability and neuronal degeneration and may contribute to neuroaxonal degeneration. These pathological changes may contribute to increased NfL release during disease progression [21]. Thus, NfL may be a marker not only of structural neuronal injury but also of more general neurobiological processes related to synaptic failure and network dysfunction [22,23].

#### 3.2.3. Neuroinflammation

Neuroinflammation is a common feature of the pathogenesis of many neurodegenerative diseases. Activated microglia and astrocytes release inflammatory mediators, reactive oxygen species, and neurotoxic cytokines that are associated with neuronal injury and may contribute to neuroaxonal damage. Chronic inflammatory responses have been associated with demyelination, mitochondrial dysfunction, and axonal degeneration, which may contribute to increased NfL concentrations [24]. Correlations between NfL concentrations and inflammatory biomarkers, such as glial fibrillary acidic protein (GFAP), soluble triggering receptor expressed on myeloid cells 2 (sTREM2), and various cytokines, suggest a close relationship between neuroinflammatory activity and neuroaxonal injury. Hence, these data highlight the importance of combining NfL with inflammatory biomarkers to understand disease mechanisms in more detail [25].

#### 3.2.4. Blood–Brain Barrier Disruption

The blood–brain barrier (BBB) regulates the movement of molecules between the central nervous system and the systemic circulation. Normally, under physiological conditions, the BBB limits the circulation of neuronal proteins to peripheral blood. However, BBB dysfunction is beginning to emerge as a feature of many neurodegenerative diseases. Reduced barrier integrity may allow CSF NfL to cross into the bloodstream, thereby contributing to elevated plasma and serum NfL concentrations. In addition to enhancing biomarker detectability, BBB disruption may be a marker of disease-related vascular pathology and neuroinflammatory processes. Therefore, circulating levels of NfL likely reflect the sum of neuronal injury, neuroinflammation, and BBB permeability alterations [26]. BBB disruption may facilitate the movement of NfL into the peripheral circulation; however, circulating NfL concentrations are influenced by multiple biological processes and should not be interpreted solely as evidence of BBB dysfunction [27].

### 3.3. Measuring Technologies

Highly sensitive analytical technologies that can detect very low concentrations of proteins have greatly expanded the clinical utility of NfL. There are several methods for NfL quantification available that have advantages and limitations in terms of sensitivity, throughput, and clinical application [5,6,16].

#### 3.3.1. ELISA (Enzyme-Linked Immunosorbent Assay)

ELISA was one of the earliest techniques used to quantify NfL. This method is based on antibody detection and offers a relatively simple and low-cost measurement of NfL levels [6]. ELISA has been extensively used in CSF studies where NfL levels are much higher than in blood. However, conventional ELISA is limited by its low analytical sensitivity, which restricts its ability to accurately detect low concentrations of NfL in plasma or serum. Therefore, its clinical utility for blood-based biomarker analysis is still limited when compared with newer technologies [6,28].

#### 3.3.2. Electrochemiluminescence (ECL) Immunoassays

Compared to traditional ELISA approaches, ECL platforms provide improved sensitivity, a wider dynamic range, and better reproducibility. ECL assays combine immunological recognition with electrochemically generated luminescent signals for accurate quantification of low-abundance proteins. Several studies have successfully used ECL-based platforms to measure NfL in CSF and blood samples. These systems offer better analytical performance and higher throughput, making them suitable for large biomarker studies [6].

#### 3.3.3. Single Molecule Array (Simoa)

Simoa technology is a major breakthrough in biomarker detection and is now the gold standard for NfL quantification in blood. Simoa is an ultra-sensitive detection method that isolates and detects single protein molecules in microscopic reaction chambers [6,8]. This enables detection at the femtomolar level. Simoa assays have significantly advanced NfL research by providing a reliable and sensitive means of quantifying circulating NfL concentrations in plasma and serum. Several studies have shown strong associations between blood and CSF NfL levels, supporting the use of minimally invasive blood-based testing for disease diagnosis, prognosis, and monitoring [6,7].

#### 3.3.4. Emerging Ultrasensitive Assays

Several next-generation technologies are in development to further improve the capabilities of biomarker detection. These include digital ELISA platforms, microfluidic immunoassays, nanomaterial-enhanced biosensors, aptamer-based detection systems, and multiplex proteomic technologies [29]. Emerging approaches aim to increase sensitivity, decrease assay costs, increase scalability, and permit simultaneous measurement of multiple biomarkers. These advances may allow for the development of integrated biomarker panels that combine NfL with inflammatory, synaptic, metabolic, and genetic markers, thus supporting comprehensive neurodegeneration profiling and precision medicine applications (Table 1) [30].

Although there have been major technological advances, further standardization of sample collection, assay calibration, analytical platforms, and reference intervals is needed to provide consistent measurement and support clinical implementation across laboratories.

### 3.4. Challenges in the Biological Interpretation and Measurement of NfL

While NfL has emerged as one of the most sensitive biomarker of neuroaxonal injury, there are a number of biological and analytical factors to consider when interpreting the clinical significance of NfL. Higher NfL concentrations indicate ongoing neuronal damage but do not identify the cause of injury. NfL should therefore be interpreted in conjunction with clinical findings, neuroimaging, and other disease-specific biomarkers rather than being considered specific to any particular disease [31]. NfL levels vary in CSF and blood. CSF typically shows higher NfL concentrations and more directly reflects central nervous system-associated pathology, whereas serum and plasma measurements are less invasive and more convenient for longitudinal monitoring [32]. Technological advances in ultrasensitive immunoassays, especially Simoa technology, have markedly improved blood NfL detection. Inter-platform variability can result from differences in analytical platforms, assay calibration, and laboratory protocols, highlighting the need for assay standardization [20].

Biological variability adds to the complexity of NfL interpretation. Higher baseline concentrations of NfL are consistently associated with older age, even in healthy individuals [33]. Other factors such as renal dysfunction, systemic inflammation, cerebrovascular disease, traumatic brain injury, peripheral neuropathy, and other neurological or medical conditions may also affect circulating NfL levels [34]. Therefore, the use of age-adjusted reference intervals and careful consideration of clinical context are essential for proper interpretation. Despite these limitations, there is scope for standardized analytical methods, consistent reference ranges, and integration of NfL with complementary molecular, imaging, and clinical biomarkers to improve its clinical utility and facilitate broader implementation in precision neurology.

### 3.5. Clinical Application of NfL (Diagnosis, Disease Progression, Response to Treatment and Prognosis)

The advent of NfL as a sensitive biomarker of neuroaxonal injury has greatly advanced the field of neurodegenerative disease diagnostics and monitoring. Rather, NfL is a general marker of neuronal injury in a wide range of neurological diseases, as opposed to disease-specific biomarkers that reflect disease-specific pathological processes [5,7]. With the availability of ultrasensitive detection technology, it is possible to reliably measure NfL in blood samples, opening new opportunities for translating research findings into clinical practice. NfL is increasingly recognized as a valuable biomarker for disease diagnosis, disease progression, therapeutic response, and clinical outcome prediction and is therefore a key biomarker in the field of precision neurology [8,15].

Although NfL is a highly sensitive marker of neuroaxonal injury, its diagnostic utility varies among neurodegenerative diseases. Because NfL reflects neuronal damage rather than disease-specific pathology, it should not be used as a stand-alone diagnostic biomarker [8,9,15]. Instead, it is most informative when interpreted together with clinical findings, neuroimaging, and disease-specific biomarkers such as amyloid-β, phosphorylated tau, α-synuclein, and GFAP. This multimodal approach improves diagnostic confidence and differential diagnosis across neurodegenerative disorders [8,35].

Monitoring disease progression is challenging as neurodegenerative diseases show great clinical and biological heterogeneity. NfL is a promising biomarker for monitoring disease over time that is strongly associated with ongoing neuroaxonal injury and disease activity [5]. The clinical evidence supporting longitudinal monitoring is strongest in multiple sclerosis, where serum NfL has become an established biomarker of inflammatory disease activity, treatment response, and future disability progression [36]. Elevated NfL levels are consistently associated with faster disease progression, brain atrophy, cognitive decline, and motor damage across the major neurodegenerative disorders. Reported studies have shown that NfL levels are often elevated before the onset of clinical symptoms and rise with disease progression, thus serving as a sensitive marker of neurodegenerative burden [8,15]. Serum NfL is an established biomarker in multiple sclerosis for monitoring disease activity, and correlates with lesion burden, relapse frequency, and future disability. Together, these findings support the utility of NfL as a dynamic biomarker for monitoring disease evolution, assessing neurodegenerative burden, and tracking progression across diverse neurological disorders [37].

Assessment of therapeutic response is key for clinical management and drug development. However, conventional clinical endpoints may take a long time to evolve and may not truly reflect biological processes. Current evidence supporting therapeutic monitoring is strongest in multiple sclerosis, where reductions in serum NfL following disease-modifying therapies closely reflect treatment effectiveness. In Alzheimer’s disease and several other neurodegenerative disorders, the use of NfL as a pharmacodynamic biomarker remains under active investigation and requires further clinical validation [7,8,15]. By contrast, chronically elevated NfL may reflect ongoing neuronal damage or an insufficient treatment response. Therefore, NfL is increasingly considered a valuable biomarker to monitor therapeutic efficacy, evaluate disease-modifying therapies, and serve as an early measure of treatment response in clinical trials to facilitate personalized treatment strategies and accelerate drug development [38,39].

In addition to its utility in diagnosis and disease monitoring, the prognostic value of NfL has been demonstrated most consistently in amyotrophic lateral sclerosis, where higher concentrations strongly correlate with disease progression and survival [36]. Although prognostic associations have also been reported in Alzheimer’s disease, frontotemporal dementia, Huntington’s disease, and Parkinson’s disease, these findings require further validation before widespread clinical implementation [21]. NfL also predicts clinical outcomes, with baseline and follow-up concentrations consistently associated with future disease progression, functional decline, and survival. Elevated NfL levels have been associated with faster cognitive and motor decline, earlier disease conversion, and worse prognosis in major neurodegenerative diseases. These results indicate the potential of NfL for risk classification, individual prognosis, and personalized clinical decision-making. Therefore, the best clinical utility of NfL will probably be achieved by combining it with multi-omics, neuroimaging, and clinical data, which will allow comprehensive neurodegeneration profiling and the advancement of precision neurology [5,7,8,15,40].

Although NfL can be measured in both CSF and blood, important differences exist between these sample types. CSF NfL is thought to influence central nervous system pathology and generally shows higher concentrations, whereas serum and plasma measurements provide a less invasive approach for longitudinal monitoring [41]. Despite advances in ultrasensitive assays such as Simoa, differences in assay platforms, calibration methods, and laboratory procedures may contribute to inter-platform variability [42]. Furthermore, biological factors, including age, renal function, systemic inflammation, recent trauma, and other neurological comorbidities, can influence circulating NfL concentrations [34]. Therefore, standardized analytical protocols, age-adjusted reference intervals, and careful clinical interpretation are essential for the reliable use of NfL in research and clinical practice.

Moreover, the level of evidence is not uniform across these applications. Serum NfL has relatively strong clinical evidence for monitoring disease activity in multiple sclerosis and for prognostic assessment in amyotrophic lateral sclerosis, while its roles in diagnosis and treatment monitoring for other neurodegenerative diseases are mostly complementary or investigational [34]. Thus, NfL should not be interpreted as a single diagnostic biomarker, but should be combined with clinical assessment, neuroimaging, and disease-specific biomarkers.

## 4. NfL in Neurodegenerative Diseases

NfL is widely recognized as a reliable indicator of neuroaxonal injury across a broad range of neurodegenerative diseases [8]. NfL concentrations are not disease-specific, but their level and changes over time usually reflect the extent of neuronal injury, disease activity, and clinical progression [15]. The advent of ultrasensitive detection technologies has made it possible to measure NfL in CSF and blood, and its application in clinical and translational research has become widespread. The following sections summarize the current evidence supporting the diagnostic, prognostic, and disease-monitoring applications of NfL across major neurodegenerative disorders, while recognizing that the strength of clinical evidence differs among diseases [5,7].

### 4.1. Alzheimer’s Disease (AD)

Alzheimer’s disease is the most common neurodegenerative disorder characterized by amyloid-β deposition, tau pathology, and progressive neurodegeneration [40]. Besides established amyloid and tau biomarkers, NfL has emerged as a sensitive marker for neuroaxonal injury and disease progression [15]. Elevated NfL concentrations in both CSF and plasma have been consistently reported in patients with AD and can be detected during preclinical and early stages of the disease, suggesting that neuroaxonal degeneration starts before the onset of overt dementia [39]. Current studies have shown strong associations between increased plasma NfL levels and hippocampal and cortical atrophy and faster cognitive decline [39,40]. Although NfL is not disease-specific, its combination with amyloid-β and phosphorylated tau markedly enhances disease staging, prognostic evaluation, and therapeutic monitoring, thereby supporting its inclusion in multimodal biomarker panels for Alzheimer’s disease [15,43]. However, because NfL is not specific to Alzheimer’s disease pathology, its clinical interpretation should always be integrated with amyloid-β, phosphorylated tau, neuroimaging findings, and cognitive assessment.

### 4.2. Parkinson’s Disease (PD)

Parkinson’s disease is defined by progressive loss of dopaminergic neurons and α-synuclein pathology. Although NfL levels are lower than those observed in diseases such as AD or ALS, increased plasma and CSF NfL concentrations are consistently associated with more severe motor problems, cognitive decline, and more rapid disease progression [44]. On the other hand, NfL has been shown to be very useful in distinguishing PD from atypical parkinsonian syndromes such as multiple system atrophy (MSA), progressive supranuclear palsy (PSP), and corticobasal degeneration (CBD), which are generally associated with higher levels of NfL in blood and CSF based on more widespread neuroaxonal damage [44,45]. Moreover, the combination of NfL with α-synuclein biomarkers, neuroimaging, and clinical assessment improves disease classification, prognostic assessment, and individualized management of parkinsonian disorders [8,45]. Current evidence indicates that NfL is particularly valuable for differentiating Parkinson’s disease from atypical parkinsonian syndromes rather than serving as a diagnostic biomarker for Parkinson’s disease itself.

### 4.3. Amyotrophic Lateral Sclerosis (ALS)

Amyotrophic lateral sclerosis is a disease in which upper and lower motor neurons progressively degenerate, causing muscle weakness, paralysis, and respiratory failure. Among the major neurodegenerative disorders, ALS has some of the highest concentrations of NfL, which reflects extensive neuroaxonal injury [46]. Elevated levels of NfL in CSF and blood can be detected early in the disease course, often before clinical diagnosis, and correlate strongly with disease severity, rate of progression, and survival [47,48]. Most studies have shown that NfL levels are higher in patients with more rapidly progressive ALS than in patients with slower disease progression. Among currently available fluid biomarkers, NfL has the strongest clinical evidence in ALS, particularly for prognostic assessment, disease progression monitoring, patient stratification, and clinical trial enrichment [47,48].

### 4.4. Multiple Sclerosis (MS)

Multiple sclerosis is a chronic inflammatory and neurodegenerative disease resulting in demyelination, axonal injury, and progressive neurological disability. NfL is among the most clinically validated biomarkers in MS, with increased levels in serum and CSF strongly correlated with inflammatory activity, lesion burden, relapse rate, and neuroimaging abnormalities [7,16]. Acute relapses are typically associated with transient increases in NfL levels, while continued elevations indicate ongoing neuroaxonal injury and an increased risk of future disability progression. Importantly, serum NfL levels decrease with effective disease-modifying therapy, supporting their utility as a biomarker of therapeutic response. Thus, NfL is increasingly used for disease monitoring and personalized treatment approaches in MS patients [16,37,38]. Among neurodegenerative and neuroinflammatory disorders, multiple sclerosis currently represents the disease in which serum NfL has achieved the greatest level of clinical validation for routine monitoring of disease activity and therapeutic response.

### 4.5. Frontal Temporal Dementia (FTD)

Frontotemporal dementia is a heterogeneous group of neurodegenerative disorders characterized by the progressive degeneration of the frontal and temporal regions of the brain. Clinical presentation includes behavioral disturbances, executive dysfunction, and language problems. Patients with FTD have consistently higher NfL concentrations in CSF and blood compared to both healthy controls and patients with psychiatric disorders presenting with similar symptoms [49,50]. Higher NfL levels correlated with greater brain atrophy, poorer physical function, and faster disease progression [51]. Importantly, NfL may help differentiate FTD from primary psychiatric disorders, a frequent diagnostic dilemma in the clinical practice. Furthermore, elevated NfL levels have been observed in asymptomatic carriers of pathogenic variants in the MAPT, GRN and C9orf72 genes, indicating a potential application for early detection of disease and risk assessment [50,51]. Nevertheless, NfL should be interpreted as a complementary biomarker and used alongside genetic testing, neuroimaging, and clinical evaluation to improve diagnostic accuracy.

### 4.6. Huntington’s Disease (HD)

**Figure 2:**
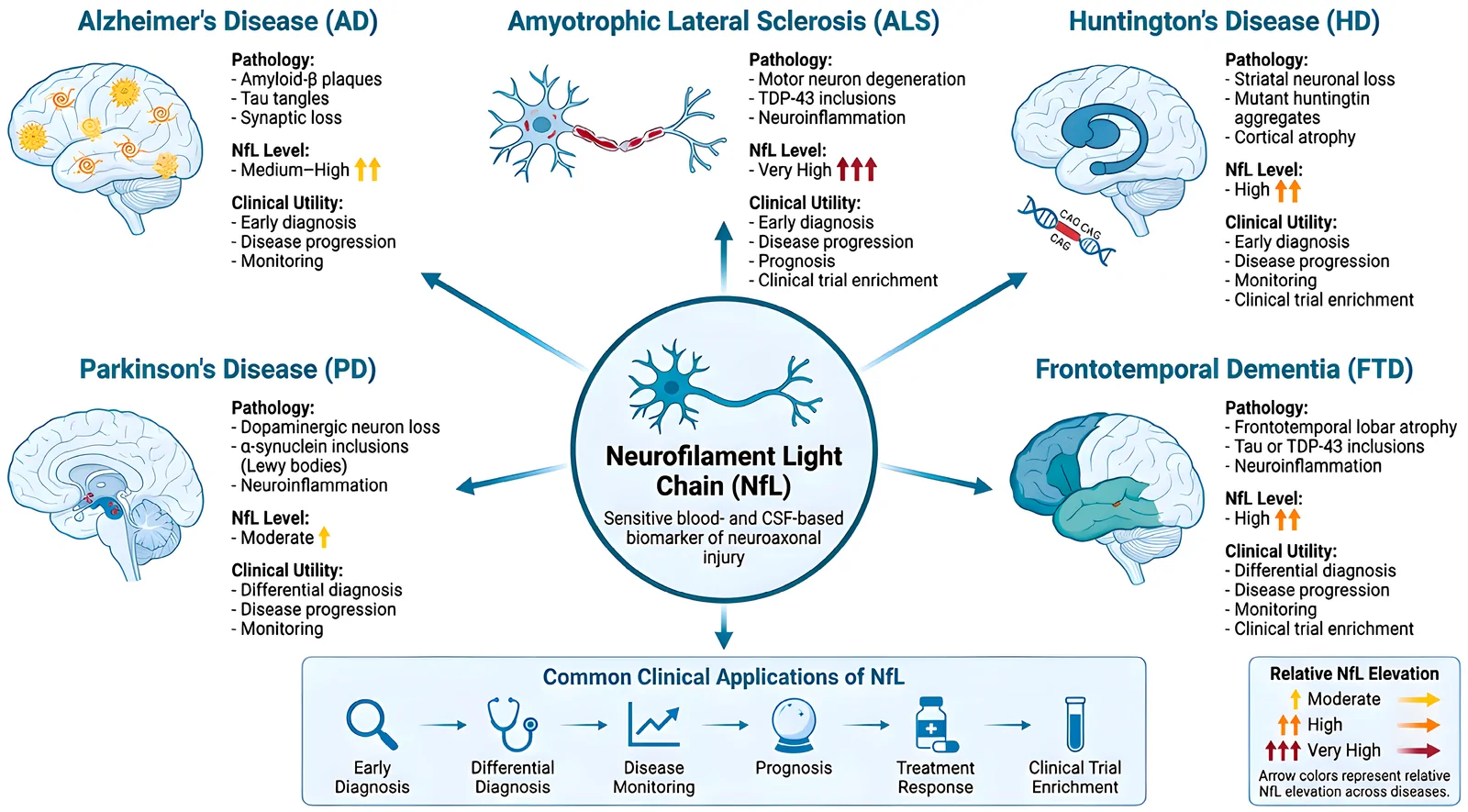
Clinical applications of neurofilament light chain (NfL) across major neurodegenerative disorders. The illustration summarizes the pathological features, relative NfL levels, and clinical applications of NfL in Alzheimer’s disease, Parkinson’s disease, amyotrophic lateral sclerosis, Huntington’s disease, and frontotemporal dementia. NfL levels increase in response to neuroaxonal injury, with the highest elevations observed in ALS. Blood- and CSF-based NfL measurements support early and differential diagnosis, disease monitoring, prognostic assessment, treatment response evaluation, and patient selection for clinical trials across neurodegenerative diseases.

Huntington’s disease is a dominantly inherited neurodegenerative disorder caused by CAG repeat expansion in the gene HTT. The disease is characterized by progressive motor dysfunction, psychiatric disturbances, and cognitive decline. Elevated NfL levels have been observed in both symptomatic and pre-symptomatic carriers of pathogenic HTT mutations [52]. Several studies have demonstrated that NfL levels increase years before the onset of clinical symptoms and are among the most promising biomarkers for identifying individuals who are approaching disease conversion. In addition, NfL levels were correlated with striatal atrophy, motor damage, and cognitive decline, suggesting their use as biomarkers of disease burden and progression. As gene-targeting therapies for HD develop, NfL may also be an important biomarker for evaluating therapeutic efficacy in future clinical trials [53,54] (Figure 2). Although these findings are encouraging, the clinical application of NfL in Huntington’s disease remains largely investigational and requires validation in larger studies that follow patients over time before routine clinical implementation.

### 4.7. NfL in Hereditary Ataxias

Hereditary ataxias are a genetically and clinically heterogeneous group of neurodegenerative disorders characterized by progressive cerebellar dysfunction and, in many cases, more widespread neuroaxonal involvement [55]. Emerging evidence suggests that NfL in blood may provide complementary information regarding neuroaxonal injury and disease progression in multiple genetic ataxias. Blood NfL concentrations were significantly higher in spinocerebellar ataxia types 1, 2, 3 and 7, Friedreich ataxia (FRDA) and ataxia-telangiectasia compared to healthy controls in the meta-analysis of 11 studies with 1006 patients and 624 healthy controls. The analysis also revealed increasing blood NfL levels with proximity to disease onset and correlation with disease severity and longitudinal disease progression in SCA3, suggesting potential value in disease evolution monitoring. However, the level and clinical relevance of NfL varied among genetic ataxias, emphasizing the necessity of disease-specific validation [56].

Friedreich ataxia is a prime example of the complexity of NfL interpretation in hereditary ataxia. Serum NfL has been reported to be elevated in individuals with FRDA compared to controls and carriers, but the association between serum NfL and clinical and genetic severity is not clear [57]. In particular, serum NfL may be relatively high in younger individuals with FRDA and decline with age, despite clinical progression of disease. This unusual pattern suggests that rather than simply tracking cumulative disease severity, NfL may reflect a combination of developmental, peripheral axonal, and neurodegenerative processes in FRDA. Therefore, although NfL could be a promising biomarker in FRDA, its clinical interpretation should take into account the age, the disease stage, and the specific biological nature of the disorder [57].

SCA3 has been a more consistent source of evidence for the potential utility of NfL. A study of 116 preclinical and manifest SCA3 individuals and 91 controls showed that serum NfL measured by the Simoa method was significantly elevated in both preclinical and manifest disease. Higher NfL was associated with lower cerebellar and brainstem volumes, and NfL concentrations correlated positively with disease stage, SARA, and ICARS scores. However, these findings should be considered promising rather than clinically validated, and further longitudinal and multicenter studies are needed before NfL can be routinely used for diagnosis, prognosis, or treatment monitoring in hereditary ataxias [58].

### 4.8. Other Neurological Diseases

Increased NfL levels have been observed in a range of neurological conditions involving neuroaxonal damage in addition to traditional neurodegenerative disorders, including traumatic brain injury, stroke, vascular dementia, prion diseases, neuromyelitis optica spectrum disorders (NMOSD), chronic inflammatory demyelinating polyneuropathy (CIDP), epilepsy, and neuroinfectious diseases [59,60]. Under these circumstances, NfL levels are generally linked to disease severity, extent of neuronal damage, and clinical outcomes, highlighting its broad utility as a biomarker of neuroaxonal injury [24]. However, since NfL is not disease-specific, its interpretation needs to be integrated with clinical findings, neuroimaging, and complementary biomarkers. Generally, the greatest clinical value of NfL comes from its use in multimodal biomarker frameworks that integrate multi-omics, neuroimaging, and artificial intelligence for comprehensive neurodegeneration profiling and precision neurology [8]. Table 2 provides a conceptual summary of the current clinical utility of NfL across major neurodegenerative and neurological disorders based on published evidence. The qualitative categories are intended to facilitate comparison and should not be interpreted as validated clinical rankings.

## 5. Multi-Omics Technologies in Neurodegeneration Research

Neurodegenerative diseases are the result of complex interactions of genetic, epigenetic, molecular, and environmental factors that cannot be explained by individual biomarkers [11,61]. Recent advances in high-throughput technologies have made it possible to comprehensively characterize these diseases by genomics, epigenomics, transcriptomics, proteomics, metabolomics, microbiome analyses, and neuroimaging [10]. Together, these complementary platforms provide important insights into disease mechanisms, biomarker discovery, and therapeutic target identification [62]. Their integration with NfL and AI provides a potent framework for complete neurodegeneration profiling and precision neurology [12,13].

### 5.1. Genomics

Genomics has greatly improved the understanding of neurodegenerative diseases by identifying genetic variants associated with disease susceptibility and progression [63]. Genome-wide association studies (GWAS) have identified key susceptibility genes like APOE, TREM2, MAPT, SNCA, and LRRK2. Whole-genome and whole-exome sequencing have identified pathogenic mutations like APP, PSEN1/2, C9orf72, and HTT [64]. In addition, polygenic risk scores (PRSs) combine multiple genetic variants to predict disease risk and could be combined with NfL and other biomarkers to improve early risk prediction and patient classification [65]. Recent studies have demonstrated that genomic information can greatly improve the clinical interpretation of NfL in combination with fluid biomarkers and clinical data. Bradley et al. (2023) conducted a genome-wide association study of plasma Alzheimer’s disease biomarkers, including NfL, in 2058 participants and found genetic variants associated with circulating NfL concentrations, suggesting inherited genetic factors contribute to inter-individual variability in NfL levels and should be taken into account in the interpretation of biomarkers [66]. Plasma NfL was measured by ultrasensitive immunoassays and genome-wide genotyping was performed to identify genetic loci associated with concentrations of the biomarker. This study showed that integrating genomic data with blood biomarkers could enhance the precision of biomarker-based risk assessment, but further validation in independent cohorts is needed. Similarly, an integrated multimodal study of 609 individuals across the Alzheimer’s disease spectrum combined plasma NfL, Aβ42/Aβ40, p-tau181, APOE genotype, and cognitive assessments to predict cerebral amyloid pathology [67]. The integrated model showed enhanced diagnostic accuracy compared to individual biomarkers alone, highlighting the complementary value of genomic information in improving the clinical utility of NfL for precision medicine. These findings indicate that genomics can provide important information on genetic susceptibility and biomarker variability and thus supports rather than replaces NfL-based assessment.

### 5.2. Epigenomics

Epigenomic changes do not change the DNA sequence, but they control the expression of genes and are important in neurodegeneration. DNA methylation, histone modifications, and chromatin accessibility regulate neuronal survival, synaptic function, and neuroinflammatory pathways [68]. Technologies such as ATAC-seq and ChIP-seq have identified disease-specific epigenetic signatures in neurons and glial cells, providing new biomarkers and possible therapeutic targets [69]. Recent studies have indicated that epigenomic profiling complements NfL, as it provides information on gene regulatory mechanisms associated with neuroaxonal injury and neurodegeneration. Smith et al. (2024) [70] performed an epigenome-wide association study (EWAS) using the Illumina Infinium HumanMethylationEPIC array on 885 blood samples to investigate DNA methylation patterns associated with 15 CSF biomarkers, including NfL. The study identified seven differentially methylated loci that were significantly associated with CSF NfL levels, suggesting that blood DNA methylation profiles may reflect biological pathways underlying neuroaxonal damage that could be used to complement NfL in biomarker interpretation [70]. Similarly, Abu Hamdeh et al. (2021) [71] performed a genome-wide DNA methylation analysis to investigate the DNA methylation of APP, MAPT, NEFH, NEFM, and NEFL genes in brain tissue from 17 patients with severe traumatic brain injury and 19 idiopathic normal pressure hydrocephalus controls. The study found significant differential methylation of the NEFL gene and several neurodegeneration-related pathways, suggesting that epigenetic changes may affect neurofilament biology after neuronal injury [71]. These results together suggest that epigenomic profiling can be a useful complementary approach to NfL in identifying regulatory mechanisms implicated in neurodegeneration, but evidence is currently limited and requires validation in larger longitudinal, multicenter studies prior to clinical application.

### 5.3. Transcriptomics

Transcriptomics gives dynamic information on gene expression during disease progression. Bulk RNA-sequencing reveals dysregulated pathways associated with inflammation, oxidative stress, and mitochondrial dysfunction, while single-cell RNA-sequencing identifies cell-type-specific molecular alterations, including disease-associated microglia and reactive astrocytes [72]. Spatial transcriptomics also preserves tissue architecture, allowing the localization of molecular changes in pathological brain areas and improving the understanding of disease development [73]. Transcriptomic studies have recently shown that profiling gene expression can complement NfL by providing mechanistic insights into the molecular pathways that contribute to neuroaxonal injury. Álvarez-Sánchez et al. (2025) [74] performed single-cell RNA sequencing analysis on peripheral blood mononuclear cells from 14 sporadic amyotrophic lateral sclerosis patients and 14 age- and sex-matched healthy controls. Plasma NfL concentrations were measured as a biomarker of neurodegeneration and correlated with transcriptomic changes in immune cell populations. The study identified disease-related changes in NK cell subsets and inflammatory signaling pathways. Multiple transcriptomic signatures were significantly associated with circulating NfL levels, suggesting that immune dysregulation contributes to neuroaxonal damage in ALS [74]. Grima et al. (2023) [75] also performed whole blood RNA sequencing on 96 sporadic ALS patients and 48 healthy controls and identified distinct molecular subtypes associated with disease heterogeneity and progression. Combining transcriptomic profiles with circulating biomarkers, such as NfL, improved the biological characterization of ALS and showed the potential of combining gene expression signatures with NfL for patient classification and biomarker discovery [75]. Although these findings show the complementary value of transcriptomics in explaining the biological processes underlying NfL elevation, validation in larger multicenter cohorts is still needed before routine clinical application.

### 5.4. Proteomics

Proteomics characterizes disease-associated protein changes in CSF and blood. CSF proteomics has identified biomarkers associated with synaptic dysfunction, neuroinflammation, and protein aggregation such as amyloid-β, tau, GFAP, YKL-40, and NfL [10,54]. Recent advances in plasma proteomics now permit minimally invasive biomarker discovery, and integration with AI has allowed the development of multi-protein panels with improved diagnostic and prognostic performance [29,62]. Recent proteomic studies have shown that the combination of NfL with large-scale protein profiling allows for a more thorough understanding of disease mechanisms and better classification of patients using biomarkers. In the largest plasma proteomic study of multiple sclerosis to date, Jacobs et al. (2024) [76] analyzed plasma samples of 407 patients with MS and 39,979 healthy controls from the UK Biobank using the Olink proximity extension assay to quantify 2911 plasma proteins. Neurofilament light chain was one of the most significantly increased proteins in MS, whereas other proteins related to immune regulation, coagulation, and neuroinflammation provided complementary information on disease severity and radiological outcomes. These findings indicated that the combination of NfL with broader proteomic signatures improves the biological characterization of MS beyond the assessment of neuroaxonal injury alone [76]. Åkesson et al. (2023) [77] also performed targeted proteomic profiling of CSF and plasma samples from 143 patients with early-stage MS and 43 healthy controls using the Olink Explore platform. NfL remained the strongest single predictor of no disease activity at two years (replication AUC = 0.77), but combining NfL with an 11-protein biomarker panel (including CXCL13, CHI3L1, TNFRSF1B, LY9 and SLAMF7) significantly improved prediction of long-term disability progression (replication AUC = 0.90). These studies highlight that proteomics complements NfL by revealing inflammatory and neurodegenerative pathways associated with disease progression, enabling better patient classification and prognostic evaluation [77]. However, these multimodal panels of biomarkers need further validation in multicenter studies before they can be used in routine clinical practice.

### 5.5. Metabolomics

Metabolomics measures downstream biochemical changes related to neurodegeneration. Alterations in energy metabolism, lipid homeostasis, and neurotransmitter pathways have been consistently reported across major neurodegenerative disorders, reflecting mitochondrial dysfunction, oxidative stress, and diminished neuronal communication [78,79]. These metabolic signatures offer functional insights into disease mechanisms and could be used together with molecular biomarkers like NfL [61]. Recent metabolomic studies have also shown that metabolic alterations add to NfL by providing functional information on the biochemical pathways related to neuroaxonal injury. In a high-resolution metabolomics study, Niedzwiecki et al. (2020) [80] analyzed plasma samples from 43 patients with AD, 45 patients with mild cognitive impairment, and 41 cognitively healthy controls, with further validation in an independent cohort of 50 AD patients and 18 controls. High-resolution metabolomics was carried out by liquid chromatography-mass spectrometry (LC-MS) to quantify plasma metabolites and identify several metabolites, including glutamine and piperine, that were significantly associated with AD diagnosis and CSF biomarkers. These findings suggest that metabolomic profiling provides complementary biochemical information to NfL by detecting metabolic disturbances associated with neurodegeneration, rather than neuroaxonal injury alone [80]. Milos et al. (2023) [81] also applied untargeted metabolomic profiling by GC-MS and LC-MS in 40 AD patients, 40 MCI patients, and 40 healthy controls, reporting significant changes in lipid metabolism, amino acid metabolism, and energy-related pathways. The study demonstrated that metabolomic signatures enhance the characterization of metabolic dysfunction related to disease and may supplement circulating NfL in evaluating disease progression and biological heterogeneity [81]. These results suggest that combining NfL with metabolomics might be important, but the evidence is mainly exploratory, and large prospective multicenter studies are needed before combined metabolomic-NfL biomarkers can be translated into routine clinical practice.

### 5.6. Microbiome Omics

The gut microbiome is increasingly recognized as a key regulator of neurological health via the gut–brain axis. Microbial dysbiosis influences immune responses, neuroinflammation, blood–brain barrier integrity, and neurotransmitter metabolism, which contribute to the pathogenesis of AD, PD, MS, and ALS. Integrating microbiome profiles with host omics data and NfL may enhance the understanding of disease heterogeneity and identify novel therapeutic opportunities [82,83]. Emerging evidence indicates that gut microbiome profiling can complement NfL by providing insights into host–microbe interactions that influence neuroinflammation and disease progression. Devolder et al. (2023) [84] performed a prospective longitudinal study, analyzing the gut microbiome by 16S rRNA gene sequencing of fecal samples, in addition to NfL levels in plasma measured by the Simoa platform, in 111 patients with multiple sclerosis. Over a median follow-up of 4.4 years, the inflammation-associated Bacteroides 2 (Bact2) enterotype was increased more than twofold in patients with increasing disability (43.6%) compared to patients with stable disease (16.1%). Interestingly, the Bact2 enterotype was more strongly associated with long-term progression of disability than plasma NfL alone, indicating that gut microbiome profiling offers complementary prognostic information beyond neuroaxonal injury biomarkers [84]. Similarly, Pauwels et al. (2026) [85] performed shotgun genome sequencing for a quantitative gut microbiome profile in a well-characterized MS cohort together with serum NfL and GFAP measurements. The study has shown significant associations between specific microbial taxa, serum NfL levels, disability status, fatigue, and other clinical features, suggesting that changes in the composition of the gut microbiota can influence biological pathways involved in neuroaxonal injury and disease progression. These findings suggest that microbiome profiling is an important complement to NfL in that it provides mechanistic information on host–microbiota interactions and neuroimmune regulation [85]. However, the evidence comes from relatively small observational cohorts, and larger sample sizes of multicenter longitudinal studies are needed before integrated microbiome-NfL biomarkers can be implemented in routine clinical practice.

### 5.7. Neuroimaging Omics

**Figure 3:**
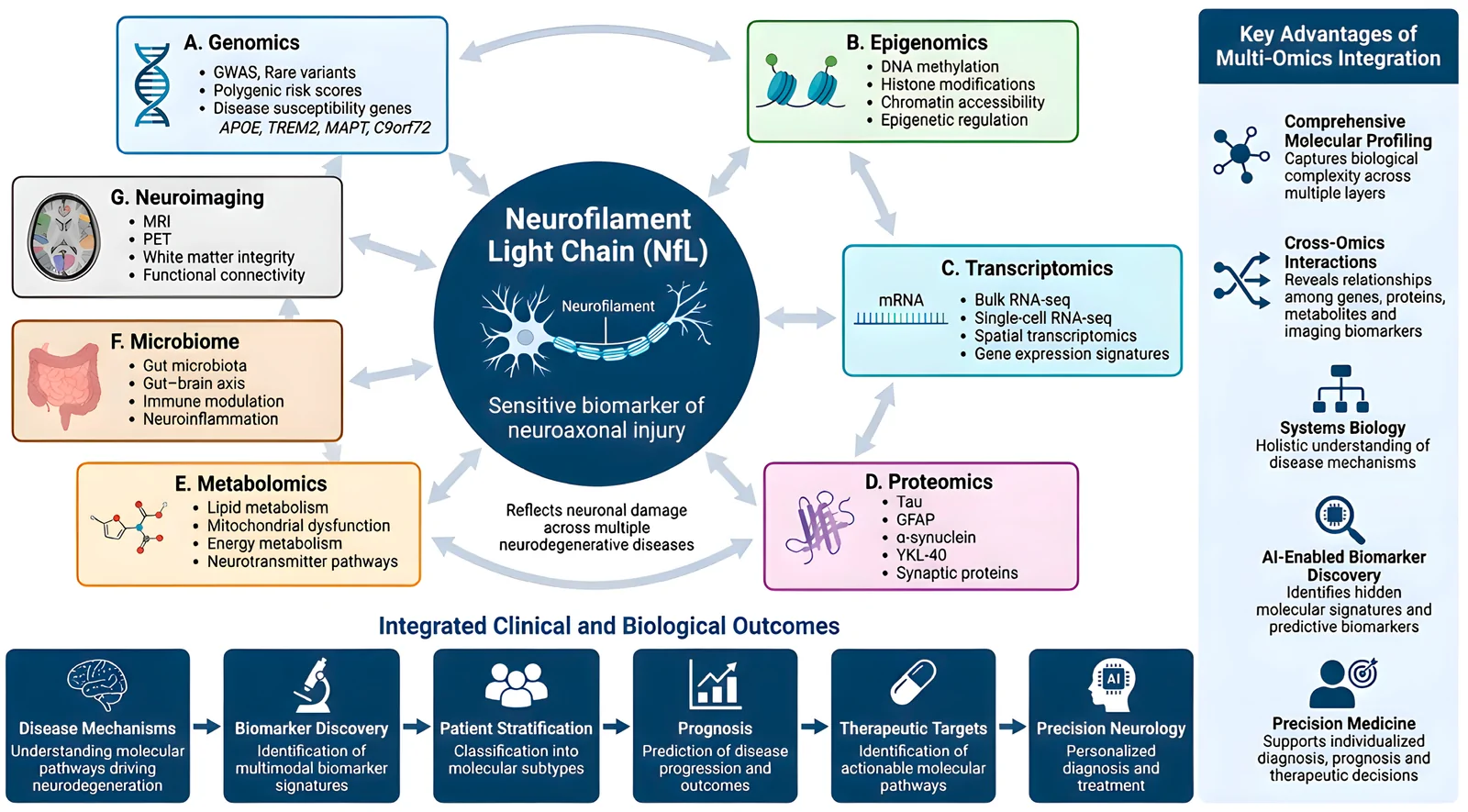
Integration of NfL with multi-omics approaches for comprehensive neurodegeneration profiling. The illustration represents how genomics, epigenomics, transcriptomics, proteomics, metabolomics, microbiome profiling, and neuroimaging provide complementary biological information to NfL. NfL provides information on neuroaxonal injury, while the different omics layers provide information on genetic, regulatory, molecular, metabolic, microbial, and structural changes associated with neurodegeneration.

Neuroimaging omics integrates advanced neuroimaging modalities with computational analyses to characterize structural and functional brain alterations. MRI allows for a detailed assessment of brain atrophy, white matter integrity, and functional connectivity [86]. PET imaging provides the opportunity to visualize molecular pathologies, such as amyloid deposition, tau accumulation, and neuroinflammation [87]. Connectomics also further characterizes large-scale neural networks and their disruption during disease progression [88]. Combining neuroimaging with NfL, multi-omics, and AI offers a comprehensive framework for tracking disease progression and enabling precision neurology [13,62] (Figure 3). Multimodal studies have recently shown that neuroimaging can be used to provide structural and functional evidence of neurodegeneration that complements NfL. Cantó et al. (2019) [89] studied 607 patients with multiple sclerosis followed for 12 years in the EPIC longitudinal multiple sclerosis cohort. Serum NfL concentrations were measured using the Simoa platform and combined with serial brain MRI and clinical assessments. Higher baseline serum NfL levels were significantly associated with disability progression and long-term brain volume loss, accounting for approximately 11.6% of the variance in brain atrophy, rising to 18.0% after adjusting for demographic and clinical factors. Together, these findings showed that adding serum NfL to MRI improves the longitudinal assessment of neuroaxonal injury and disease progression [89]. Similarly, Buchmann et al. (2023) [90] investigated the association between serum NfL and quantitative MRI measures in 167 patients with multiple sclerosis. Serum NfL levels were measured using the Simoa assay, and structural MRI was used to quantify global and regional brain atrophy. Higher serum NfL levels were significantly associated with lower brain volume, higher physical disability, and faster neurodegeneration, highlighting the complementary value of combining fluid biomarkers with neuroimaging. Together, these studies show that NfL is a sensitive marker of ongoing neuroaxonal injury, while neuroimaging defines its anatomic distribution and structural consequences, thus improving disease monitoring and prognostic assessment [90]. However, more multicenter studies are required to standardize multimodal imaging-biomarker frameworks before routine clinical implementation.

Overall, the studies presented in this section show that the different omics layers provide information that complements NfL, including genetic predisposition, regulatory mechanisms, and molecular, metabolic, microbial, and structural changes. These complementary datasets form the biological basis for further multimodal and AI-based analyzes discussed in the following sections (Table 3) [13,62].

## 6. Integrative Interpretation of NfL and Multi-Omics Data

NfL is a marker of neuroaxonal injury, and multi-omics approaches provide complementary information on the genetic, regulatory, cellular, molecular, and metabolic processes involved in neurodegeneration [8,15,29]. The studies discussed in the above section demonstrate that genomics can provide information on genetic susceptibility, transcriptomics can identify disease-associated cellular and immune pathways, proteomics can capture inflammatory and disease-specific protein signatures, metabolomics can characterize biochemical alterations, microbiome profiling can provide information on host-microbe interactions, and neuroimaging can define the structural and functional consequences of neuroaxonal injury [10,61,62]. Thus, the strength of combining NfL with multi-omics does not come from the repetition of measuring neuroaxonal injury but rather from integrating complementary biological information [13,62]. Such integration may enhance biological interpretation and support multimodal modeling, although most combined biomarker frameworks are still investigational and will require further validation [12,62]. Table 4 represents a few published studies integrating NfL with different multi-omics approaches.

## 7. Artificial Intelligence for Neurodegeneration Profiling

The rapid expansion of multi-omics technologies, neuroimaging platforms, and digital health systems has led to large volumes of biological and clinical data in neurodegenerative disease research [62,91]. These datasets contain important information on disease susceptibility, molecular mechanisms, progression trajectories, and therapeutic responses. But their complexity also poses great analytical challenges, often beyond the reach of conventional statistical methods [91]. AI, including ML, deep learning (DL), and advanced computational modeling techniques, is a transformative tool to extract meaningful insights from high-dimensional biomedical datasets [13,14,91]. AI-based methods have shown remarkable potential in recent years for integrating genomics, transcriptomics, proteomics, metabolomics, neuroimaging, and fluid biomarkers such as NfL [13,91]. The following sections focus on AI methods that have been applied or are under investigation for NfL-containing multimodal biomarker models, with a focus on their methodological strengths, limitations, and current level of evidence.

### 7.1. Why AI Is Relevant to NfL-Based Multimodal Modeling

AI is relevant to NfL-based multimodal modeling, since studies of neurodegeneration are increasingly combining fluid biomarkers, multi-omics, neuroimaging, and clinical variables with different scales, structures, and temporal characteristics. These data may have non-linear relationships and large numbers of correlated features that require appropriate feature selection, multimodal integration, and validation. In this context, AI offers analytical approaches that can be complementary to conventional statistical approaches for finding patterns and interactions. The clinical value of AI-based NfL models depends on strong validation, external testing, and reproducibility in independent populations.

Biological systems are characterized by highly complex and non-linear interactions between genes, proteins, metabolites, cells, and environmental factors. It is well established that neurodegenerative diseases involve multiple interconnected pathways that could act synergistically or antagonistically over periods of time [91]. AI algorithms can capture non-linear relationships and higher-order interactions among variables, unlike traditional linear models [91,92]. This is crucial for understanding the disease-driving mechanisms, discovering biomarker signatures, and developing predictive models able to capture the complexity of neurodegenerative disorders [91,92].

Recent studies have shown that may improve the diagnostic value of NfL by combining it with complementary fluid biomarkers, neuroimaging, genetic data, and clinical variables. Mazzeo et al. (2024) [93] studied 140 people with subjective cognitive decline, mild cognitive impairment, and Alzheimer’s disease dementia in a longitudinal study. Plasma NfL was measured using the Simoa platform and correlated with cerebrospinal fluid biomarkers, amyloid PET, 18F-fluorodeoxyglucose PET, and cognitive assessments in the A/T/N framework. The integrated prediction model had good diagnostic performance in identifying AD pathology (AUC = 0.82) and accurately predicted progression from SCD to MCI and from MCI to AD dementia over longitudinal follow-up [93]. Mattsson et al. (2017) [40] also analyzed plasma NfL in 570 participants of the Alzheimer’s Disease Neuroimaging Initiative (ADNI), including cognitively normal individuals, patients with MCI, and AD dementia. Plasma NfL concentrations were measured with the Simoa assay and integrated with CSF biomarkers, structural MRI, and cognitive measures. The multimodal approach revealed that plasma NfL was significantly correlated with neurodegeneration, brain atrophy, and cognitive decline, achieving a diagnostic accuracy of AUC = 0.87 for differentiating AD dementia from cognitively healthy individuals [40]. These studies demonstrate that AI-assisted multimodal biomarker models improve diagnostic performance by integrating NfL with other biological and imaging information complementary to NfL rather than treating NfL as a single biomarker. However, further external validation in larger multicenter cohorts is required before these models can be routinely used in clinical practice.

### 7.2. Machine Learning Approaches

Machine learning is a major subset of AI that deals with building algorithms that can learn patterns from data and predict without being directly programmed [91,92]. Machine learning approaches can be generally categorized into supervised learning, unsupervised learning, and deep learning methodologies [14,91,92].

#### 7.2.1. Supervised Learning

In supervised learning, algorithms learn from labeled datasets (where the outcomes or disease states are known). These models learn the relations between the input variables and the target outcomes, which enables them to predict and classify new samples [14,91,92].

#### 7.2.2. Random Forest (RF)

Random Forest (RF) is one of the most popular supervised machine-learning algorithms for biomarker discovery due to its ability to efficiently process high-dimensional datasets, identify important variables, and model complex non-linear interactions [94,95]. Although RF has been extensively used for classifying neurodegenerative diseases using multimodal biomarkers, studies assessing RF models specifically based on NfL are limited. There is presently no evidence that plasma NfL is used as an independent predictor, but rather as part of multimodal biomarker panels alongside amyloid-β, phosphorylated tau, APOE genotype, neuroimaging, and cognitive variables. The integrated approaches showed improved diagnostic performance and patient classification over single biomarkers alone, emphasizing the potential value of RF for multimodal NfL-based biomarker modeling [93]. However, more studies specifically evaluating the contribution of NfL within RF models are required before its independent clinical utility can be established.

#### 7.2.3. Extreme Gradient Boosting (XGBoost)

Extreme Gradient Boosting (XGBoost) is an ensemble machine-learning algorithm that has been widely used in biomedical research due to its high predictive performance, ability to efficiently handle complex datasets, and its capacity to handle missing data [96]. XGBoost has been increasingly used in neurodegenerative disease research to develop multimodal prediction models, combining fluid biomarkers, neuroimaging, genetic information, and clinical variables. But there are still few studies that specifically evaluate XGBoost models for NfL. Current evidence suggests that plasma or serum NfL is typically used as one component of multimodal biomarker panels, including amyloid-β, phosphorylated tau, APOE genotype, neuroimaging features, and cognitive assessments to improve disease classification and prediction of cognitive decline. These integrated models perform better than single-biomarker models and highlight the complementary role of NfL in AI-based multimodal models [97]. Further studies are needed to estimate the independent contribution of NfL to XGBoost models and to validate these methods in larger and more heterogeneous clinical cohorts.

#### 7.2.4. Support Vector Machines (SVMs)

Support vector machines have been applied with NfL-based multimodal modeling, particularly to combine fluid biomarkers with neuroimaging and clinical measures [91,95]. Brummer et al. (2022) [98] evaluated 152 patients with early multiple sclerosis and used support vector regression (SVR) to evaluate whether serum NfL alone or in combination with MRI measures could predict cognitive performance. Combining serum NfL, lesion volume, and gray matter volume achieved a cross-validated accuracy of 88.7% in the first cohort and 90.8% in a replication cohort of 101 early MS patients, outperforming single- and dual-biomarker models. These results suggest that SVM-based methods can augment the prognostic value of NfL with complementary measures of structural brain damage [98]. More recently, Zhu et al. (2024) [99] assessed 173 patients with Parkinson’s disease with clinical variables, structural MRI, resting-state functional MRI, and plasma NfL. The best SVM model combining clinical measures, resting-state functional MRI, and NfL among 29 multimodal classifiers achieved 76.2% mean accuracy, an AUC of 0.840, a sensitivity of 74.5%, and a specificity of 78.3% in distinguishing Parkinson’s disease with mild cognitive impairment from Parkinson’s disease with normal cognition [99]. These results suggest the potential of NfL to be incorporated in a multimodal biomarker framework rather than as a stand-alone diagnostic marker by SVM-based models.

#### 7.2.5. Unsupervised Learning

Unsupervised learning algorithms, on the other hand, identify patterns and structures in unlabeled datasets, unlike supervised algorithms. These approaches are especially useful for the discovery of disease subtypes and the characterization of biological heterogeneity [13,14,91].

#### 7.2.6. Clustering

Clustering algorithms group people based on similarities in molecular, imaging, or clinical features. Common approaches include tree-based clustering, k-means clustering, and density-based methods. In neurodegenerative disease research, clustering analyses have identified distinct molecular and clinical subgroups, which are associated with variations in disease progression, response to therapy, and prognosis. Combining NfL with multi-omics biomarkers may further improve the identification of biologically meaningful patient clusters [14,91]. Unsupervised machine-learning approaches have recently been shown to identify clinically relevant subgroups when integrating NfL with complementary biomarkers. Willard et al. (2025) [100] developed a Subtype and Stage Inference (SuStaIn) model that combined serum NfL (sNfL) with MRI-derived measures in patients with multiple sclerosis. The model was trained on 189 patients with relapsing-remitting or secondary progressive MS and externally validated on 445 newly diagnosed patients. Results found two biologically distinct MS subtypes based on the timing of elevation of sNfL and MRI abnormalities. The combined MRI-sNfL model demonstrated stronger correlations between data-derived disease stages and Expanded Disability Status Scale scores than MRI-only models in the training cohort (Spearman’s ρ = 0.420 vs. 0.231) and in the external test cohort (ρ = 0.163 vs. 0.067). Early-sNfL subtype was also associated with higher risk of new lesion formation (hazard ratio = 2.44, 95% CI: 1.38–4.30), highlighting the utility of NfL-informed clustering for identifying biologically distinct disease trajectories [100]. Hence, results provide direct evidence that NfL can help with AI-based patient stratification by providing information about neuroaxonal injury that is not captured by MRI alone. Nevertheless, before such data-driven subtypes can be routinely used in clinical practice, validation in independent disease cohorts is required.

#### 7.2.7. AI-Based Identification of Disease Subgroups 

Patient stratification involves dividing patients into clinically relevant subgroups that share biological features. AI-based stratification strategies are increasingly being used to identify disease endotypes and targets for precision medicine. Combining NfL with genomic, transcriptomic, and proteomic datasets may help identify patients with distinct neurodegenerative trajectories, potentially leading to personalized therapeutic strategies and better clinical trial design [14,91].

#### 7.2.8. Deep Learning

Deep learning is a more advanced subset of machine learning that uses artificial neural networks with multiple hidden layers to model complex relationships in large datasets [13,14,91].

#### 7.2.9. Convolutional Neural Networks (CNNs)

Convolutional Neural Networks are particularly good at analyzing image data. CNN architectures learn hierarchical feature representations automatically from MRI, PET, and histopathological images. CNN models have been shown to achieve high accuracy in detecting AD, PD, and MS from neuroimaging data in neurodegenerative diseases. Combining imaging features from CNNs with NfL and multi-omics biomarkers might substantially increase the disease classification performance [13,14,101]. Neuroimaging data has been increasingly analyzed by CNNs to capture complex spatial patterns associated with neurodegeneration. Importantly, recent work has begun to link imaging measures derived from CNNs to circulating NfL, providing a multimodal framework for interpreting structural brain changes within the framework of biochemical evidence of neuroaxonal injury. Zhang et al. (2024) [102] developed an interpretable 3D CNN model using T1-weighted MRI data from a heterogeneous dataset of 1464 participants to estimate brain age in subjects with mild traumatic brain injury (mTBI) and healthy controls. The model predicted brain age in 154 healthy controls with a mean absolute error of 3.08 years and a Pearson correlation coefficient of 0.97, and showed consistent performance across different centers [102]. In mTBI patients, the brain predicted-age gap was significantly elevated and related to cognitive impairment and increased plasma NfL concentrations. Thus, although NfL was not directly used as an input to the CNN in this work, its correlation with the CNN-derived brain-age metric illustrates how deep-learning-derived neuroimaging features can be associated with an independent fluid biomarker of neuroaxonal injury [102]. This complementary approach may improve biological interpretation of imaging-derived measures; however, further studies directly incorporating NfL into CNN models are needed to determine whether combined models provide improved diagnostic or prognostic performance.

#### 7.2.10. Recurrent Neural Networks (RNNs)

Recurrent Neural Networks are built to analyze sequential and longitudinal data, incorporating temporal dependencies into predictive models. This ability makes RNNs particularly appropriate to study disease progression and biomarker trajectories. Applications include prediction of cognitive decline and rates of disease progression, as well as modeling changes in NfL concentrations and other biomarkers over time [101]. RNNs are well-suited for longitudinal clinical data because of their capability to model temporal changes in multiple variables measured at different time points. A direct application of NfL in the CENTER-TBI cohort was reported by Bhattacharyay et al. (2023) [103], where RNN models with long short-term memory (LSTM) and gated recurrent unit (GRU) architectures were developed to predict 6-month functional outcome after traumatic brain injury (TBI). The prospective cohort comprised 1550 patients from 65 centers, and the models used 1166 pre-ICU and ICU variables, including serial NfL measurements, intracranial pressure, neurological variables, imaging findings, and other clinical data. Patient data were represented in 2 h time windows, enabling the RNN to update the predicted Glasgow Outcome Scale–Extended (GOSE) prognosis during the entire ICU stay. The full variable set explained 52% (95% CI: 50–54%) of the ordinal variance in 6-month functional outcome; dynamic ICU information contributed up to 5% (95% CI: 4–6%). Importantly, the study explicitly incorporated NfL in the longitudinal model, demonstrating the feasibility of employing NfL in temporal AI models instead of a single baseline biomarker [103]. However, the study also showed that the majority of the explained outcome variance was explained by static admission information, and the model performance was worse in patients with longer ICU stays [103]. Therefore, while RNN-based modeling offers a promising framework for integrating serial NfL measurements with clinical and imaging data, further studies will be needed to assess the additional predictive value of NfL specifically and to validate these models in independent cohorts.

#### 7.2.11. Transformers

Recently, transformer architectures have revolutionized AI research with their ability to process large-scale multimodal datasets and capture long-range dependencies. Originally developed for natural language processing, transformers are now being adapted to genomics, proteomics, and biomedical imaging applications. Multimodal transformer models are promising candidates for future precision neurology platforms, as they can simultaneously integrate NfL measurements, omics profiles, neuroimaging data, and clinical information [14,101]. Transformer-based models have also been explored more and more to tackle multimodal and longitudinal neurological data, because the attention mechanism is capable of capturing relationships across multiple kinds of data and time points. However, direct evidence of transformer models specifically incorporating NfL versus conventional machine-learning and recurrent approaches remains limited. Recent transformer studies in neurodegenerative disease have largely focused on combining neuroimaging, clinical, and genetic information, with NfL typically being viewed as a complementary fluid biomarker rather than a core input into the model. Recent multimodal transformer frameworks for Alzheimer’s disease have combined structural MRI with clinical and genetic features and reported strong internal diagnostic performance, but these studies have not measured the additional predictive value of NfL [104]. Emerging multi-omics transformer approaches are similarly beginning to integrate proteomic, metabolomic and genomic information to predict disease, although their direct application to NfL-based modeling is still at an early stage. Therefore, transformers should be regarded as a promising future framework for the integration of longitudinal NfL measurements with multi-omics, neuroimaging and clinical data, rather than a well-established NfL-specific modeling approach. Future research should specifically assess whether attention-based multimodal models provide a clinically relevant improvement over simpler models and be subject to independent external validation before translation to the clinical application.

### 7.3. Explainable AI

Many AI models are “black-boxes” which accounts for their predictive power but also limits their acceptance in clinical settings. Explainable artificial intelligence (XAI) aims to enhance model transparency by pinpointing the components that affect predictions and enabling biological interpretation [105,106].

#### 7.3.1. SHapley Additive Explanations (SHAP)

SHAP is one of the most popular explainability frameworks for explaining machine learning models. It measures the contribution of each feature to a given prediction by calculating how much each feature influences the results. SHAP analyses have also been employed in neurodegenerative disease studies to identify critical biomarkers that contribute to classification and prediction of the disease. SHAP can be applied to multimodal datasets to identify the relative importance of NfL, genetic variants, imaging features, and other biomarkers in predictive models [105]. SHAP can improve the interpretability of NfL-based ML models by quantifying the contribution of individual biomarkers and clinical or imaging features to model predictions. A relevant example was reported by Zhu et al. (2024) [99] in a study with 173 individuals with Parkinson’s disease (PD), in which multimodal machine-learning models were developed to distinguish between patients with PD and mild cognitive impairment (PDMCI) and those with PD and normal cognition (PDNC). Models included demographic and clinical variables, plasma biomarkers (including NfL and GFAP), and structural and functional MRI measures. The best support vector machine model integrating clinical variables, resting-state functional MRI, and plasma NfL among 29 multimodal classifiers reached a mean accuracy of 0.762, an area under the curve of 0.840, sensitivity of 0.745, and specificity of 0.783 in the validation set. SHAP analysis was then used to interpret the model and showed that higher plasma NfL concentrations contributed to an increased predicted risk of PDMCI, in addition to clinical and neuroimaging features [99]. This example shows how SHAP can be used to identify the contribution of NfL in a multimodal model, as opposed to considering NfL as an isolated diagnostic marker. SHAP values, however, are indicators of feature contribution to a model prediction and should not be interpreted as proof of biological causality. Further validation needed to confirm that SHAP-based explanations are consistent across independent cohorts and model architectures.

#### 7.3.2. Local Interpretable Model-Agnostic Explanations (LIME)

LIME gives local explanations to individual predictions by approximating complex models with simpler interpretable models. This approach helps clinicians and researchers to understand why a specific prediction was made. LIME has been used increasingly to interpret AI-driven biomarker models and facilitate the clinical adoption of machine learning systems. Combining LIME with multimodal models focused on NfL may increase trust, reproducibility, and regulatory acceptance [105,106]. LIME is a post hoc explainable AI technique that generates local explanations for individual predictions by approximating the behavior of a complex model in the vicinity of a specific observation. LIME has been applied in neurodegenerative disease research, mostly for the interpretation of classification models on imaging and clinical data but sparsely on NfL-based biomarker modeling. A systematic review of explainable AI applications in Alzheimer’s disease found LIME to be a significant method for interpreting machine-learning predictions, but the existing studies mainly concentrated on neuroimaging and clinical features, not multimodal biomarker models including NfL [107]. Therefore, LIME could potentially be used to help explain the contribution of an individual patient’s NfL level to a multimodal prediction together with imaging, clinical, and multi-omics variables, but this application is yet to be adequately validated. Future studies should test the stability and clinical usefulness of LIMEs in NfL models and compare them with other interpretability approaches like SHAP.

### 7.4. Federated Learning (FL)

A significant challenge in neurodegenerative disease research is the need for large, diverse, and geographically distributed datasets. Centralizing patient information is generally constrained by data-sharing limitations, privacy concerns, and regulatory requirements [106]. FL is a privacy-preserving framework that allows multiple institutions to collaborate in training machine-learning models without the need to transfer individual patient data to a central repository. This is specifically true for neurodegenerative diseases where large and heterogeneous datasets of fluid biomarkers, neuroimaging, clinical information, and multi-omics measurements are often distributed across hospitals and research centers. FL studies have demonstrated the feasibility of collaborative MRI analysis across institutions in multiple sclerosis. For example, Hindawi et al. (2025) [108] tested a federated nnU-Net model on 512 MRI cases across three clinical sites for automated MS lesion segmentation without the need to share raw patient data. The federated model achieved Dice scores of 0.66–0.80 in held-out test sets, but differences in performance were observed between sites due to differences in the underlying datasets [108]. Similarly, Bai et al. (2024) [109] showed federated MS lesion segmentation on clinical sites with noise-resilient training. This work emphasizes the potential of distributed learning to tackle inter-center heterogeneity. However, these studies did not include NfL or other fluid biomarkers [109]. Therefore, direct evidence for FL on NfL is still limited, and the use of FL for distributed NfL, multi-omics, imaging, and clinical datasets should be viewed as an emerging research direction rather than an established clinical approach. Future studies should explore the possibility of integrating serial NfL measurements with imaging and multi-omics data within federated multimodal models while preserving patient privacy, and evaluate model performance across independent institutions and diverse patient populations.

#### 7.4.1. Multi-Center Collaboration

Federated learning allows multiple institutions to collectively train AI models without having to share sensitive patient data. Local models are trained separately, and model parameters are exchanged and combined. This approach allows large-scale international collaborations between hospitals, research institutes, and biobanks while respecting data ownership and privacy regulations. Federated learning has the potential to greatly improve the development of reliable NfL-based predictive models by utilizing heterogeneous patient populations [110,111].

#### 7.4.2. Privacy-Preserving Analytics

Preserving privacy is an important consideration in biomedical AI. Federated learning combined with secure aggregation, differential privacy, and encrypted computation allows for collaborative analysis while reducing risks of data exposure. With the exponential growth of multi-omics and clinical datasets, privacy-preserving AI frameworks will become increasingly important for the development of globally applicable systems for neurodegeneration profiling. Integration of NfL, omics data, and federated learning may ultimately enable the establishment of large-scale precision neurology networks that can accelerate biomarker discovery and clinical translation [13,14,92,105].

AI technologies together provide the computational basis to transform complex multi-omics and biomarker datasets into clinically actionable knowledge. The combination of NfL with genomics, transcriptomics, proteomics, metabolomics, neuroimaging, and clinical data offers AI-based approaches remarkable opportunities for enhancing diagnosis, prognosis, patient stratification, and therapeutic decision-making in neurodegenerative diseases (Table 5) [13,14].

## 8. Multimodal NfL-Based Biomarker Modeling: Integration and Validation

The development of NfL-based multimodal models involves several methodological steps, including preprocessing of data, feature selection, multimodal data fusion, model development, and validation. These steps define how efficiently information from NfL, multi-omics, neuroimaging, and clinical variables can be integrated, avoiding overfitting and ensuring interpretability.

### 8.1. Data Standardization

Data standardization is the backbone of AI-driven multi-omics integration, providing a reliable basis for the comparison of datasets generated using different technologies, protocols, and patient cohorts [112,113]. Data standardization strategies like quality control, normalization, batch-effect correction, and missing data imputation are applied to minimize technical variability while preserving biologically meaningful signals [113]. Data standardization is especially relevant in NfL-centric studies, as biomarker measurements might be produced from different analytical platforms, such as ELISA, electrochemiluminescence, and Simoa assays [113]. AI-based data standardization pipelines further optimize the integration of NfL with multi-omics and neuroimaging data, improving reproducibility, multicenter comparability, and the reliability of predictive models [112,113].

### 8.2. Feature Selection

The high dimensionality of multi-omics data calls for effective feature selection to reduce noise and computational complexity, prevent overfitting, and identify biologically relevant variables [91,95]. Compared with traditional statistical methods, AI-based methods can better consider the complex interactions between biomarkers and improve prediction performance and biological interpretability [91,112]. Popular methods include Random Forest feature importance, Recursive Feature Elimination (RFE), Least Absolute Shrinkage and Selection Operator (LASSO), Boruta algorithms, SHAP-based feature ranking, and deep learning attention mechanisms [91,95,105,112]. Combined with NfL, these approaches reveal biomarker signatures related to neuroinflammation, synaptic dysfunction, mitochondrial dysfunction, and neuroaxonal degeneration, offering better disease prediction and mechanistic insights than individual biomarkers alone [13,91,113].

### 8.3. Multi-Modal Data Fusion

Multi-modal data fusion combines different biological and clinical data into one analytical framework to comprehensively characterize neurodegenerative diseases [91,113]. AI-based fusion strategies integrate NfL with genomics, transcriptomics, proteomics, metabolomics, neuroimaging, and clinical data to improve biomarker discovery and predictive modeling [13,91,113].

Early (feature-level) fusion combines NfL, omics, and imaging features into a single dataset prior to model training, allowing machine learning algorithms to capture multilevel biological interactions [113]. This approach improves disease classification and biomarker discovery but can be affected by high dimensionality, feature scale differences, and missing data [91,113].

In intermediate fusion, modality-specific representations are learned first by separate machine learning or deep learning models, and then they are fused into a shared feature space [91,113]. This strategy often leads to better predictive performance when integrating complex datasets like NfL, transcriptomics, and neuroimaging, by keeping modality-specific information while enabling cross-modal interactions [14,91,113].

**Figure 4:**
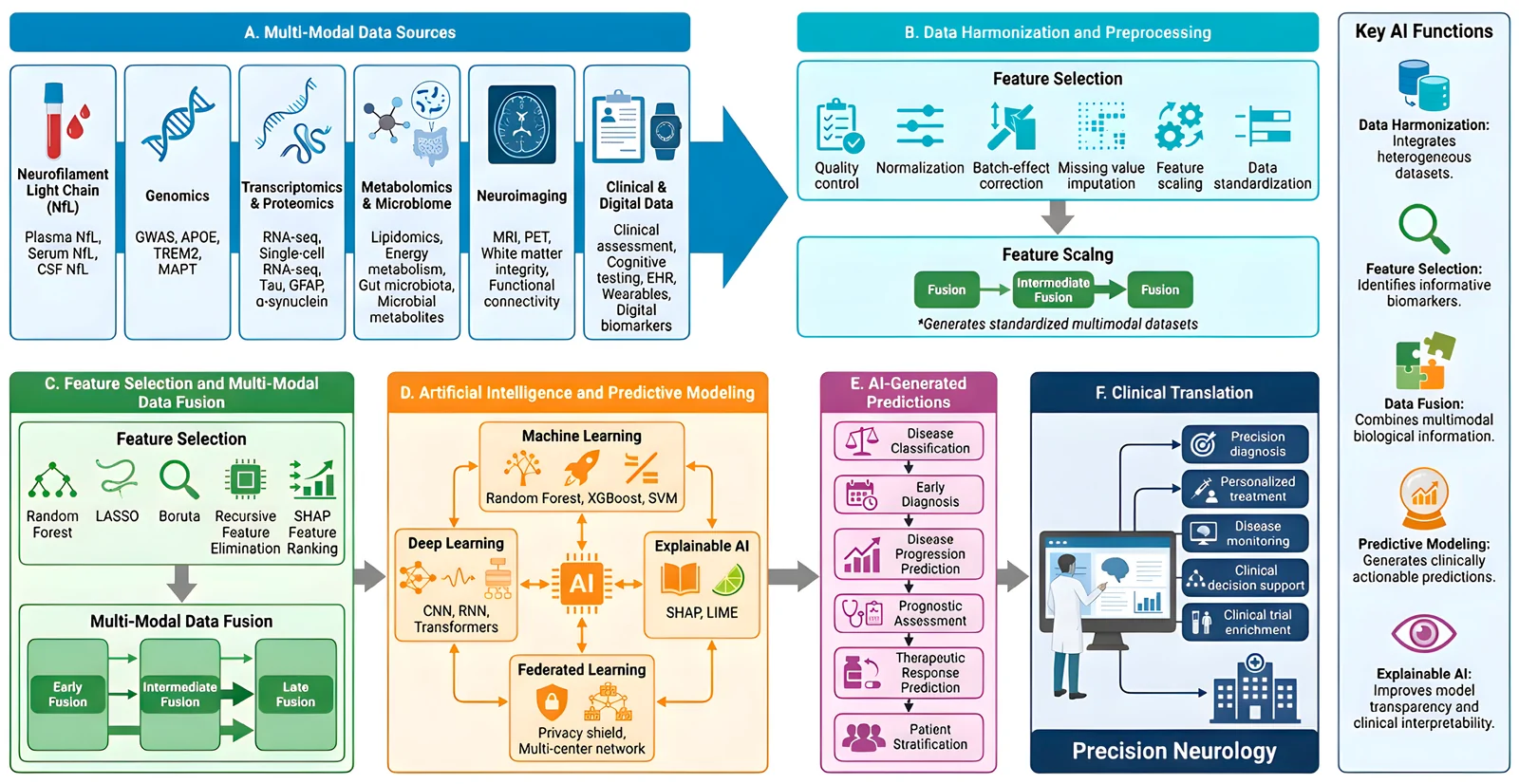
AI-based integration workflow of NfL with multi-modal biomarker data. The framework comprises data harmonization, feature selection, multimodal data fusion, model development, explainability, and validation. NfL can be combined with multi-omics, neuroimaging, and clinical variables to develop models of multimodal biomarkers.

Late (decision-level) fusion merges predictions from models trained on different modalities of data independently [113]. This approach is more flexible, interpretable, and reliable to missing data, making it especially suitable for multicenter studies with heterogeneous datasets [91,113] (Figure 4). Hybrid frameworks combining early, intermediate, and late fusion are more and more adopted to maximize predictive accuracy while keeping biological interpretability [113] (Table 6).

### 8.4. Disease Classification

The integration of NfL with multi-omics and neuroimaging data via AI-driven methods is an emerging approach for disease classification. These multimodal models have shown promising performance in research cohorts in several studies, but have not been adequately validated for routine clinical diagnosis in diverse populations [91,92,113]. Machine learning algorithms such as Random Forest, XGBoost, Support Vector Machines, and deep neural networks have consistently outperformed single-modality approaches in diagnostic accuracy [13,91,96]. Because NfL is a non-specific indicator of neuroaxonal injury, AI-based disease classification models achieve their greatest clinical value when NfL is integrated with disease-specific molecular biomarkers, neuroimaging features, and clinical data rather than being used in isolation [13,91,113]. For example, the combination of plasma NfL with phosphorylated tau, APOE genotype, and MRI characteristics improves the ability to distinguish cognitively normal individuals, mild cognitive decline, and AD, while similar multimodal approaches improve the ability to distinguish PD from atypical parkinsonian syndromes [12,39,114].

### 8.5. Progression Prediction

A major aim of neurodegenerative disease research is to predict disease progression. The clinical courses vary widely between individuals and are difficult to predict with traditional approaches [13,91]. NfL measurements collected over time are used in AI-based progression models together with multi-omics and imaging data to predict future disease trajectories. Measurements of NfL have been combined with multi-omics and imaging data and used in AI-based progression models to study future disease trajectories. Preliminary studies suggest that such models may improve prediction of cognitive decline, motor decline, disability, and survival. However, their performance and clinical utility need further validation in independent and multicenter cohorts [13,101,113]. Longitudinal machine learning frameworks such as Recurrent Neural Networks (RNNs), long short-term memory (LSTM) networks, and transformer-based architectures are particularly powerful in modeling temporal dynamics of biomarkers [14,101]. Such approaches capture complex interplay of disease-related variables and provide personalized prediction of disease progression [101,113].

For AD, the combination of NfL trajectories with transcriptomic and neuroimaging biomarkers has been reported to improve the prediction of conversion from mild cognitive impairment to dementia [113,114]. Comparable approaches have been successfully applied to predict progression rates in ALS, MS and HD [13,91,113]. If prospectively validated, such progression models could aid earlier risk assessment, patient counseling and clinical trial design [13,91,113].

### 8.6. Therapeutic Response Prediction

The use of AI to incorporate NfL with multi-omics and clinical data may offer a framework to study therapeutic response, though this use is largely investigational [13,91,113]. NfL has demonstrated utility as a pharmacodynamic and disease monitoring biomarker in selected settings, especially multiple sclerosis, but the use of multimodal AI models to predict individual treatment response needs more prospective validation. [13,91]. Similar approaches are being pursued in other neurodegenerative diseases, but for now should be considered as research applications and not as validated tools for treatment selection [113].

Future NfL-centric precision neurology platforms may have the ability to combine longitudinal biomarker measurements with multi-omics, imaging, and clinical data to support personalized treatment decisions. However, these adaptive treatment frameworks are still conceptual and require prospective clinical validation, standardized measurements, regulatory qualification, and demonstration of clinical benefit before implementation in routine practice (Table 7) [13,91,113].

## 9. Clinical Applications and Precision Neurology

The combination of NfL, multi-omics technologies, and AI provides a novel platform to study disease biology and to develop multimodal biomarker models [91]. NfL has shown clinical utility in specific applications, notably for monitoring disease in multiple sclerosis and prognostic assessment in certain neurodegenerative diseases, but the broader integration of NfL with multi-omics and AI is still largely in the research stage. The following sections therefore distinguish established or well-supported applications from emerging and prospective uses [13,91,113].

Precision neurology seeks to move beyond symptom-based disease classification towards biologically informed management of patients [13,113]. When sufficiently validated, integrated NfL, multi-omics, imaging and clinical models could aid in biologically informed disease profiling. For this reason, the following sections focus on the possible clinical implications and current evidence, rather than reiterating the modeling approaches described in the above section.

### 9.1. Early Diagnosis

One of the major challenges in neurodegenerative diseases is early diagnosis, since pathological changes often begin years or even decades before the appearance of clinical symptoms [7,19,113]. Conventional diagnostic tools are comparatively less able to detect the considerable neuronal damage that may have accumulated in these pre-clinical stages [13,19]. NfL is a promising biomarker for early neuroaxonal injury and has been reported to be raised in the presymptomatic stages of AD, HD, FTD and ALS [19,47,50,51]. However, NfL alone is not specific enough for accurate early diagnosis [7,15].

NfL may contribute to early identification of neuroaxonal injury when combined with disease-specific biomarkers and other molecular or imaging measures. However, because NfL is not disease-specific, multimodal approaches for early diagnosis remain primarily investigational and require further validation before routine clinical use [13,24,39,113]. Genomic risk factors such as APOE, TREM2, and MAPT variants can be used to identify individuals with an increased susceptibility, and transcriptomic and proteomic biomarkers can provide insights into the molecular changes that are taking place [39,49,115]. AI algorithms can combine these datasets to identify disease-associated patterns that cannot be detected with traditional analytical methods [13,14,60]. This type of multimodal framework may help identify high-risk individuals before irreversible neurodegeneration, allowing for earlier treatment and possibly better therapeutic outcomes [13,113].

### 9.2. Differential Diagnosis

Accurate differential diagnosis is important for selecting appropriate therapeutic strategies and predicting clinical outcomes [91,113]. However, overlapping symptoms among neurodegenerative disorders often complicate diagnostic evaluation. PD can be difficult to differentiate from atypical parkinsonian syndromes like progressive supranuclear palsy, multiple system atrophy, and corticobasal degeneration [91]. Similarly, frontotemporal dementia can be misdiagnosed as a psychiatric disorder. AD shares many clinical features with vascular dementia and dementia with Lewy bodies [113].

AI-guided multimodal biomarker models including NfL, disease-specific proteins, neuroimaging markers, and genetic information can significantly improve diagnostic accuracy [91,113]. Higher NfL levels together with α-synuclein biomarkers may help to differentiate PD from atypical parkinsonian disorder, while the inclusion of NfL with amyloid-β, phosphorylated tau, and APOE genotype may improve the classification of AD [114]. By integrating multiple biological dimensions simultaneously, AI-based systems can identify disease-specific molecular signatures and allow more accurate differential diagnosis than any single biomarker [91,113].

### 9.3. Disease Monitoring

Neurodegenerative diseases continue to change over time, with biological and clinical changes continuing over time. Thus, effective disease monitoring is important to evaluate disease progression, assess the efficacy of treatment, and guide clinical management. Among the fluid biomarkers available today, NfL is especially suited for longitudinal follow-up as concentrations mirror ongoing neuroaxonal damage [7,15,24]. Several studies have shown associations between NfL levels and brain atrophy, cognitive decline, functional decline, and disease activity [39,40,47,50,51]. Serial NfL measurements combined with transcriptomic, proteomic, metabolomic, and neuroimaging data allow for a more comprehensive monitoring of the disease course [113]. AI algorithms can detect temporal biomarker trajectories and early biological changes before clinical manifestations [91,101]. AI-powered analysis of serial NfL and multimodal data could ultimately lead to better longitudinal monitoring of disease. However, using such models for real-time treatment adaptation is a prospective application that requires clinical validation [113].

### 9.4. Prognostic Modeling

A major challenge in neurodegenerative medicine is the prediction of future disease trajectories [91]. The clinical course is highly variable among patients even within the same diagnostic group, and thus prognosis is difficult on an individual basis [13,113]. Prognostic models based on AI combine NfL measurements with multi-omics and neuroimaging biomarkers to predict future clinical outcomes. These models can predict cognitive decline, motor impairment, increase in disability, risk of institutionalization, and survival [101,113].

In AD, elevated plasma NfL in combination with amyloid, tau, and imaging biomarkers has been associated with accelerated progression from mild cognitive decline to dementia [43,114]. Similar prognostic relationships have been described in ALS, MS, HD, and FTD [13,47,50,51,113]. AI-enabled prognostic models can provide personalized risk estimations of disease progression that could improve patient counseling, support care planning, and facilitate clinical decision-making across disease management [13,91,113]. While NfL has demonstrated prognostic associations in a number of neurodegenerative diseases, AI-based multimodal prognostic models are still primarily research tools. Prospective validation, calibration in different populations, and demonstration that model-based predictions improve clinical decision-making will be required for their clinical utility.

### 9.5. Patient Stratification

Neurodegenerative diseases show significant biological heterogeneity resulting in different disease mechanisms, progression rates, and responses to treatments among patients. This complexity is often not reflected in traditional diagnostic categories [13,113]. Patient classification aims to identify biologically distinct subgroups based on molecular and clinical features. By using AI-based clustering and machine learning approaches, NfL can be integrated with genomics, transcriptomics, proteomics, and imaging data to identify disease endotypes that may be hidden from conventional clinical assessments [91,113].

For example, patients with AD may have distinct molecular profiles with varying contributions of amyloid pathology, tau pathology, neuroinflammation, or vascular dysfunction [114]. Similarly, the disease courses in ALS and MS patients might be heterogeneous and linked to certain biomarker signatures [47,51,116]. Identification of biologically distinct subgroups may improve understanding of disease heterogeneity and clinical-trial design. However, whether AI-derived NfL-based subgroups can reliably inform individualized treatment remains to be determined [113].

### 9.6. Clinical Trial Enrichment

The failure rate of clinical trials in neurodegenerative disease is high, largely due to biological heterogeneity, late diagnosis, and poor patient selection. NfL has emerged as a promising biomarker for selecting patients for clinical trials, reflecting active neuroaxonal injury and disease progression and allowing the identification of patients most likely to benefit from disease-modifying therapies [13,18,28,116]. Further improvement in participant selection by integration of NfL with multi-omics biomarkers and AI allows the identification of disease-specific molecular signatures that improve cohort homogeneity and statistical power [13,113]. NfL is being more and more investigated as a biomarker for patient selection and pharmacodynamic assessment in clinical trials. Integration with multi-omics and AI may further enhance participant characterization and trial classification. However, these multimodal approaches are still investigational and need prospective validation before they can be routinely used for clinical trial enrichment [6,18,113].

Overall, the combination of NfL, multi-omics technologies, and AI presents a promising research framework for disease characterization, assessment, patient classification, and clinical-trial design. However, the extent to which these integrated approaches improve clinical outcomes remains to be established through prospective, multicenter, and externally validated studies [13,91,113].

## 10. Challenges and Limitations

The remarkable advances in NfL research, multi-omics technologies, and AI-driven analytics have not yet succeeded in overcoming the scientific, technical, clinical, and regulatory challenges that limit their widespread clinical implementation. While integrated biomarker frameworks offer great promise to improve diagnosis, prognosis, and personalized treatment strategies, key limitations remain in biological interpretation, data quality, reproducibility, scalability, and ethical governance [91,113,117]. Addressing these challenges is critical for translating AI-enabled NfL-centered multi-omics platforms from research settings into routine clinical practice [91,113].

### 10.1. Biological Variability of NfL

A major limitation of NfL is its non-specificity for disease. Although high NfL levels are reliable markers of neuroaxonal injury, they are not specific to the cause and are elevated in numerous neurodegenerative and neurological disorders [7,15]. Furthermore, physiological factors, including age, sex, body mass index, renal function, and systemic inflammation, influence the levels of circulating NfL, leading to interindividual variability [7,8]. Disease stage and progression also further complicate interpretation, with some disorders characterized by gradual increases in NfL and others by acute elevations during periods of rapid neuroaxonal damage [15,47,51]. Thus, universal reference ranges are still difficult to establish. For clinical utility, NfL should be interpreted with respect to demographic factors, disease-specific biomarkers, and clinical findings [7,15]. Integration with multi-omics data may further improve its biological specificity and disease characterization [13,113].

### 10.2. Data Standardization Issues

Standardization remains a challenge for integrating NfL with multi-omics and neuroimaging data [112,113,118]. Technical differences resulting from sample collection and storage conditions, analytical platforms, and bioinformatics pipelines limit reproducibility and cross-study comparisons [112,113]. Similarly, variable measurements can occur due to different NfL detection methods like ELISA, electrochemiluminescence, and Simoa assays, emphasizing the need for standardized testing methods [113]. Differences in scanner specifications, acquisition protocols, and image-processing workflows also affect neuroimaging data and can reduce model generalizability [86,112,113]. Standardized operating procedures, reference materials, and consensus reporting guidelines will be essential to improve data consistency, enabling reliable multimodal biomarker integration and facilitating clinical translation [86,112,113,118].

### 10.3. Multi-Center Reproducibility

Reproducibility is critical for the clinical translation of NfL and multi-omics biomarkers integrated with AI [113]. Despite promising results reported, many findings are not yet sufficiently validated in independent cohorts and diverse populations [91,113]. Differences in patient demographics, disease characteristics, healthcare practices, and environmental factors can have a significant impact on biomarker performance, often reducing the applicability of models developed in single-center studies [91,113]. Moreover, AI algorithms, especially deep learning models, may learn cohort-specific patterns instead of biologically relevant signatures when trained on limited datasets [14,91,105]. Large-scale, multi-center studies using standardized protocols, international collaborations, and federated learning approaches will be critical to improve model reliability, reproducibility, and clinical applicability, while preserving patient privacy [14,91,105,110].

### 10.4. Small Sample Sizes

Limited sample size is a major challenge in neurodegenerative disease research, especially for rare disorders and for large-scale multi-omics studies where data generation is costly and resource-intensive [113,117]. The small size of the cohorts limits the reliability and generalizability of the AI models, increases the risk of false-positive findings, and reduces statistical power [113]. In particular, high-dimensional multi-omics datasets are prone to overfitting, especially in the absence of external validation [91,112]. These difficulties are even more acute in rare disorders, such as HD, inherited forms of frontotemporal dementia and inherited ALS, where patient numbers are intrinsically low [50,51,113]. Generating datasets with sufficient statistical power and improving the reliability of biomarker discovery and AI-driven predictive models will be essential and will require the expansion of international data-sharing initiatives, biobanks and collaborative research networks [113].

### 10.5. Data Privacy and Ethics

The integration of genomic, multi-omics, imaging and clinical data raises major ethical and privacy issues, as these datasets often contain highly sensitive personal and genetic information [91,110,113]. AI-based analyses require access to large-scale patient data, which increases the risks of unauthorized access, re-identification, and data misuse [91,113]. Furthermore, AI models that are trained on non-representative datasets can produce algorithmic bias, resulting in poorer performance in diverse populations and increasing differences in healthcare outcomes [91,117]. Another key challenge is model interpretability, since the limited transparency may hinder clinical adoption [91,105,113,117]. Explainable AI methods such as SHAP and LIME improve model interpretability but need to be further refined for routine clinical use [105,106,113]. Future precision neurology frameworks must therefore balance technological innovation with strong ethical governance, data security, transparency, and responsible management of patient data [13,91,113].

### 10.6. Regulatory Challenges

Regulatory approval remains a key challenge for clinical implementation of AI-driven multimodal biomarker platforms [117,119]. Adaptive AI systems are not static diagnostic tests; they change as new data flows in, so there are challenges in terms of algorithm transparency, validation, updating software, and monitoring performance post-deployment [117,119]. Likewise, the clinical translation of multi-omics biomarkers requires clear demonstration of analytical validity, clinical validity, and clinical utility via large-scale prospective studies [112,117,119]. NfL in combination with multi-omics and AI adds to the regulatory complexity, as it merges multiple analytical technologies into a single framework [113,117,119]. Hence, there is a need for standardized validation pathways and consistent regulatory guidelines [117,119]. Continued collaboration among researchers, clinicians, industry, and regulatory agencies, as well as advances in biomarker standardization, explainable AI, and federated learning, will be critical to enable safe and effective clinical translation of AI-integrated precision neurology [13,91,113,117] (Table 8).

## 11. Future Perspectives

The integration of NfL, multi-omics technologies and AI has already transformed the landscape of neurodegenerative disease research [13,91,113]. Current biomarker frameworks, however, still only capture part of the complex biological processes involved in neurodegeneration [91,113]. Emerging technologies will further refine resolution, scalability, and clinical utility of neurodegeneration profiling by allowing more precise characterization of cellular heterogeneity, spatial organization, longitudinal disease dynamics, and individualized treatment responses [72,120]. The convergence of next-generation omics platforms, digital health technologies, foundation AI models, and computational disease simulations is likely to drive future developments [72,120,121]. Combined, these innovations could transform neurology from reactive disease management to predictive, preventive, and personalized healthcare [13,91,121].

### 11.1. Single-Cell Multi-Omics

In contrast, conventional bulk omics approaches average molecular signals across heterogeneous cell populations, potentially hiding cell-type-specific modifications involved in neurodegeneration [72,122]. In contrast, single-cell multi-omics allows for simultaneous analysis of genomic, epigenomic, transcriptomic, and proteomic profiles at single-cell resolution, revealing disease-associated changes in neurons, microglia, astrocytes, and other brain cell populations [72,122,123]. Combining single-cell multi-omics and NfL can help to identify the cellular sources of neuroaxonal injury and improve understanding of disease mechanisms [72]. Moreover, the analysis of these high-resolution datasets using AI is expected to allow the discovery of new biomarkers, improve disease classification, and help develop precision therapeutics [13,72,122].

### 11.2. Spatial Omics

Single-cell omics identifies cellular heterogeneity but often loses the spatial context of tissue organization [72,124]. This limitation is bypassed by spatial omics, which preserve the anatomical distribution of gene and protein expression in intact tissues [72,124,125]. Spatial transcriptomics, spatial proteomics, and multiplex imaging are valuable for gaining insight into localized pathological features such as amyloid plaques, tau tangles, Lewy bodies, and neuroinflammatory lesions [72,124]. The combination of spatial omics with NfL, neuroimaging, and AI has the potential to generate comprehensive molecular maps that link regional pathology to systemic changes in biomarkers, thereby enhancing our comprehension of disease progression and allowing for more accurate characterization of neurodegeneration [72,124,125].

### 11.3. Digital Biomarkers and Wearables

Advances in digital health technologies now enable continuous and non-invasive monitoring of neurological function via wearable sensors, smartphones, and remote monitoring devices [120,126]. These platforms provide real-time data on movement, cognition, sleep, speech, and other physiological parameters, and objective and sensitive measures of disease progression that may detect early changes before clinical symptoms appear [91,120,126]. The integration of digital biomarkers, NfL, multi-omics data, and AI has the potential to improve disease monitoring, prognostic modeling, and treatment assessment [91,120,126]. The ongoing assessment of digital health data holds potential to facilitate early detection of disease progression, personalized therapeutic interventions, and adaptive clinical management in neurodegenerative disorders [91,120].

### 11.4. Foundation Models and Generative AI

Foundation models and generative AI are significant advances in AI, allowing for large-scale integration and analysis of multimodal biomedical data [120,121]. Foundation models are distinct from traditional ML models because they are trained on massive datasets and can be applied to genomics, transcriptomics, proteomics, neuroimaging, and clinical data. These models have the potential to integrate NfL, multi-omics, neuroimaging, electronic health records, and digital biomarkers in a unified framework in neurodegeneration research, improving biomarker discovery, disease prediction, and patient classification. Moreover, generative AI may accelerate drug discovery, identify novel therapeutic targets, and enable AI-assisted clinical decision-making, thereby advancing precision neurology [120,121].

### 11.5. Digital Twins in Neurology

Digital twins are computational models that use biological and clinical data from the real world that are constantly updated to simulate disease progression [121,127]. In neurology, digital twins could integrate NfL measurements, multi-omics profiles, neuroimaging, digital biomarkers, and clinical data to build personalized models of neurodegenerative diseases. Such virtual representations can predict disease trajectories, anticipate therapeutic responses, and evaluate treatment strategies prior to clinical implementation [127]. Digital twin technology is still in its infancy but has the potential to improve disease modeling, optimize personalized treatment planning, and accelerate precision neurology through individualized predictive healthcare [121,127].

### 11.6. Precision Neurology Ecosystems

**Figure 5:**
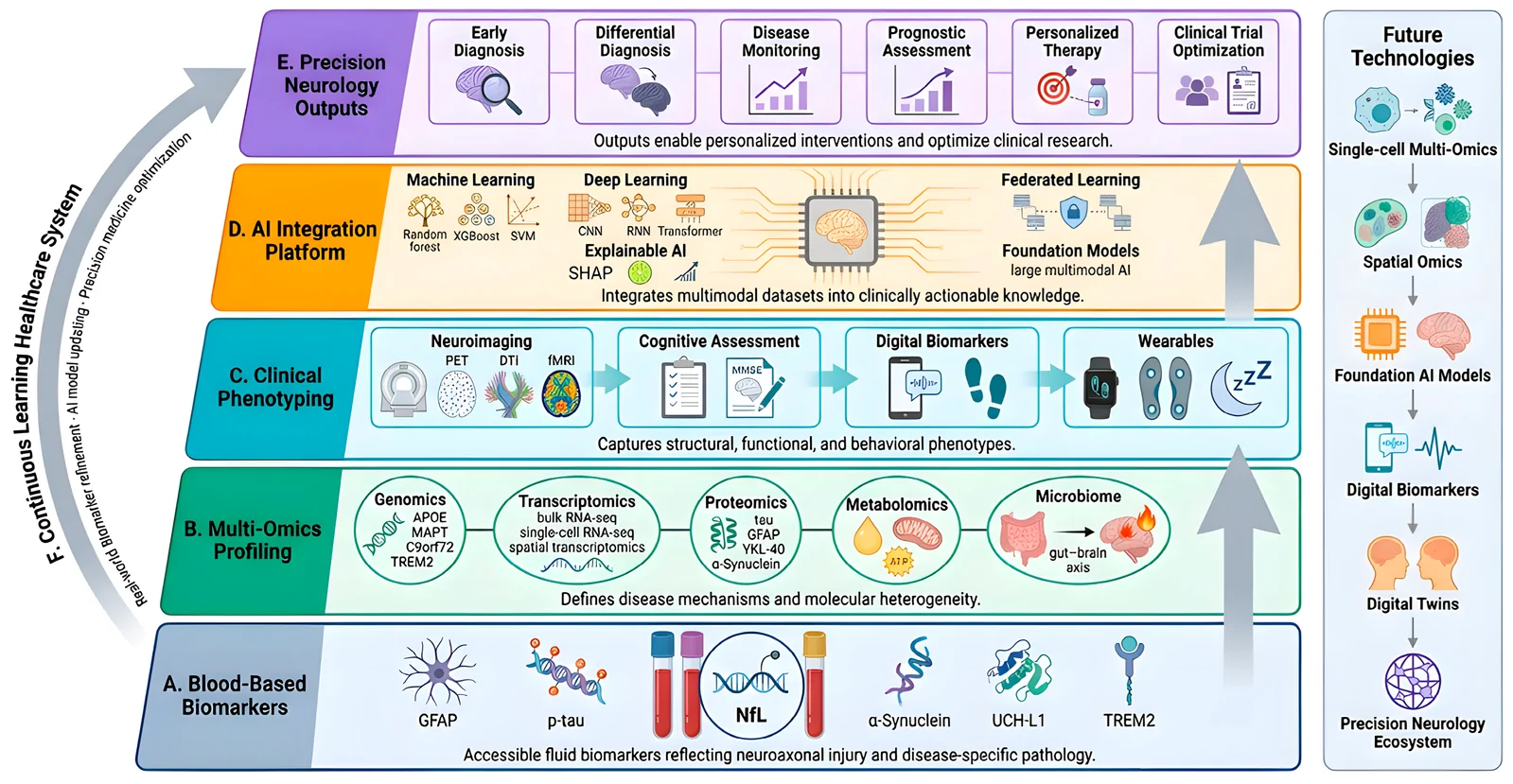
Proposed AI-enabled framework for NfL-based precision neurology. The framework shows a potential for NfL to be integrated with multi-omics, clinical, and neuroimaging data. Established and emerging applications are distinguished, with AI-based disease classification, progression prediction, patient classification, therapeutic-response modeling, and clinical-trial optimization considered as developing research applications requiring further validation.

The future of neurodegenerative disease management relies on integrated precision neurology ecosystems that combine blood-based biomarkers, multi-omics technologies, neuroimaging, digital biomarkers, electronic health records, and AI-driven clinical decision-support systems [91,121]. Future precision-neurology ecosystems may combine NfL with multi-omics, neuroimaging, digital biomarkers, and clinical data. However, the clinical utility of such integrated systems has yet to be established through prospective validation, standardized data collection, and demonstration of benefit in real-world clinical settings [13,91,113,121]. In this context, NfL is expected to be a major biomarker of neuroaxonal injury, and multi-omics will provide mechanistic understanding, while AI will translate heterogeneous data to clinically meaningful information. Emerging technologies such as single-cell and spatial omics, digital biomarkers, foundation AI models, and digital twins are expected to characterize a new era of precision neurology, accelerating biomarker-guided clinical trials and enabling personalized management of neurodegenerative diseases [72,120,121,127] (Figure 5).

## 12. Conclusions

Neurodegenerative diseases remain major clinical challenges because of their complex pathogenesis, biological heterogeneity, overlapping symptoms, and limited availability of effective disease-modifying therapies. Neurofilament light chain is an essential component of the neuronal cytoskeleton and one of the most sensitive fluid biomarkers of neuroaxonal injury. Although NfL has demonstrated broad clinical utility across neurodegenerative diseases, its level of clinical validation varies among different disorders. The strongest evidence currently supports its application in monitoring disease activity and treatment response in multiple sclerosis and in prognostic assessment of amyotrophic lateral sclerosis, whereas its use in Alzheimer’s disease, Parkinson’s disease, frontotemporal dementia and Huntington’s disease is best considered complementary to disease-specific biomarkers, neuroimaging findings and clinical evaluation. Its concentrations in cerebrospinal fluid and blood provide valuable information for disease detection, longitudinal monitoring and prognostic assessment, while its roles in disease detection, therapeutic-response evaluation and clinical trial stratification vary across disorders and remain under investigation in several clinical settings. However, NfL should not be interpreted as a stand-alone diagnostic biomarker because it reflects the extent of neuroaxonal injury rather than disease-specific molecular pathology. Because NfL reflects the extent of neuronal injury rather than a disease-specific molecular mechanism, its greatest value lies in its integration with complementary molecular, imaging, digital, and clinical biomarkers. The combined application of NfL, multi-omics technologies, neuroimaging, and artificial intelligence provides a promising systems-level framework for characterizing the biological complexity of neurodegeneration. AI-driven multimodal models have shown potential to integrate heterogeneous datasets for disease classification, progression prediction, identification of biologically distinct patient subgroups, and evaluation of therapeutic response, but most of these applications remain at the research and validation stage. Importantly, AI-based models may provide greater clinical value when NfL is integrated with disease-specific biomarkers rather than used in isolation. However, the clinical benefit of such multimodal approaches requires prospective and external validation. These approaches can also help connect neuroaxonal injury with genetic susceptibility, transcriptional dysregulation, protein alterations, metabolic disturbances, neuroinflammation, structural brain changes, and functional decline. Emerging technologies, including single-cell and spatial multi-omics, digital biomarkers, wearable devices, foundation models, federated learning, and digital twins, may further improve disease profiling and longitudinal prediction, but their application to NfL-based clinical decision-making remains largely investigational. Nevertheless, successful implementation will require standardized NfL measurement, harmonized multi-omics and imaging protocols, large-scale multicenter validation, transparent and explainable AI models, representative patient populations, and powerful data-governance frameworks. Overall, NfL-centered multimodal biomarker systems offer a promising strategy for comprehensive neurodegeneration profiling. Future research should focus on disease-specific clinical validation of NfL across different neurodegenerative disorders and on establishing standardized multimodal biomarker frameworks that integrate NfL with molecular, imaging, and clinical data. Continued advances in multi-omics integration, computational modeling, analytical standardization, and prospective clinical validation will be essential to determine whether these approaches can improve diagnostic accuracy, prognostic assessment, therapeutic monitoring, and personalized management of neurodegenerative diseases.

## Figures and Tables

**Table 1:** Comparison of NfL detection technologies for neurodegenerative disease biomarker assessment.

Detection Technology	Principle	Sample Type	Sensitivity	Advantages	Limitations	Clinical Applications
ELISA	Antibody-based immunoassay	CSF, Serum	Moderate	Simple, cost-effective	Lower sensitivity	Research purpose
Electrochemiluminescence (ECL)	Electrochemically induced luminescence	CSF, Plasma	High	Improved dynamic range	Specialized instrumentation	Biomarker quantification
Single Molecule Array (Simoa)	Digital immunoassay	Plasma, Serum, CSF	Ultra-high	Detects very low NfL concentrations	Expensive	Early diagnosis, monitoring
Automated Immunoassays	Automated antibody detection	Serum, Plasma	High	High throughput	Platform variability	Clinical laboratories
Emerging Ultrasensitive Assays	Novel biosensor-based methods	Blood, CSF	Very High	Rapid detection, point-of-care potential	Limited validation	Future clinical applications

**Table 2:** Conceptual summary of the current clinical utility of NfL across major neurodegenerative and neurological disorders.

Disease	NfL Level	Diagnostic Utility	Prognostic Utility	Treatment Monitoring	Major Clinical Application	Current Evidence
Alzheimer’s disease	↑	Moderate	Excellent	Moderate	Complementary biomarker with Aβ/pTau	Emerging/Complementary
Parkinson’s disease	↑	Moderate	Good	Moderate	Differentiation from atypical parkinsonism	Emerging
Amyotrophic lateral sclerosis	↑↑↑	Excellent	Excellent	Excellent	Established prognostic biomarker	Strong
Multiple sclerosis	↑↑	Excellent	Excellent	Excellent	Established prognostic biomarker	Strong
Frontotemporal dementia	↑↑	Good	Excellent	Moderate	Complementary prognostic biomarker	Moderate
Huntington’s disease	↑↑	Good	Excellent	Moderate	Emerging biomarker requiring validation	Emerging
Traumatic brain injury	↑↑	Good	Good	Moderate	Injury severity and prognosis	Moderate
Stroke	↑↑	Moderate	Good	Moderate	Prognosis and functional outcome	Moderate

**Note:** The qualitative categories (Excellent, Good and Moderate) are presented as conceptual summaries based on the available published evidence and are not formal clinical rankings or guideline recommendations. ↑, ↑↑, and ↑↑↑ indicate increasing level of NfL from low to moderate to marked elevation, respectively.

**Table 3:** Major multi-omics technologies and their applications in neurodegenerative diseases.

Omics Platform	Biological Information	Representative Biomarkers	Clinical Applications	Integration with NfL
Genomics	Genetic susceptibility	APOE, TREM2, MAPT, C9orf72	Risk prediction	Identifies genetic predisposition
Epigenomics	Gene regulation	DNA methylation, Histone modifications	Disease mechanisms	Explains regulatory changes
Transcriptomics	Gene expression	mRNA, ncRNA	Pathway analysis	Links NfL with gene activity
Proteomics	Protein expression	Tau, GFAP, YKL-40, α-synuclein	Biomarker discovery	Complements neuronal injury
Metabolomics	Metabolic alterations	Lipids, amino acids	Functional assessment	Identifies metabolic dysfunction
Microbiome	Gut microbial composition	Bacterial taxa	Gut–brain axis	Neuroinflammation
Neuroimaging	Structural and functional changes	MRI, PET, Connectomics	Disease staging	Correlates with NfL levels

**Table 4:** Representative published studies integrating NfL with multi-omics biomarkers in neurodegenerative diseases.

Omics Layer	Disease	Cohort (*n*)	Sample Type	Analytical Method	Integration with NfL	References
Genomics	Alzheimer’s disease	2058	Plasma	GWAS + plasma NfL	Genetic variants associated with	[66]
	Alzheimer’s disease	609	Plasma	APOE genotyping + plasma biomarkers	NfL + APOE + Aβ42/Aβ40 + p-tau181	[67]
Epigenomics	Alzheimer’s disease	885	Blood DNA + CSF	EWAS (Illumina EPIC array)	DNA methylation associated with CSF NfL	[70]
	Traumatic brain injury	17 TBI/19 controls	Brain tissue	Genome-wide DNA methylation	Differential methylation of NfL	[71]
Transcriptomics	Amyotrophic lateral sclerosis	14 ALS/14 controls	PBMC + Plasma	scRNA-seq + Simoa	NK-cell transcriptomics correlated with plasma NfL	[74]
	Amyotrophic lateral sclerosis	96 ALS/48 controls	Whole blood	RNA-seq	Molecular subtypes integrated with NfL	[75]
Proteomics	Multiple sclerosis	407 MS/39,979 controls	Plasma	Olink PEA	NfL integrated with 2911 proteins	[76]
	Multiple sclerosis	143 MS/43 controls	CSF and Plasma	Olink Explore	11-protein panel + NfL	[77]
Metabolomics	Alzheimer’s disease	43 AD/45 MCI/41 HC	Plasma	LC-MS	Metabolites associated with AD biomarkers	[80]
	Alzheimer’s disease	40 AD/40 MCI/40 HC	Plasma	GC-MS and LC-MS	Metabolic pathways complement NfL	[81]
Microbiome	Multiple sclerosis	111	Stool + Plasma	16S rRNA sequencing + Simoa	Gut microbiome + plasma NfL	[84,85]
Neuroimaging	Multiple sclerosis	607	Serum + MRI	Simoa + serial MRI	MRI + NfL	[89]
	Multiple sclerosis	167	Serum + MRI	Simoa + MRI volumetry	Brain volume correlated with NfL	[90]

**Table 5:** Overview of AI approaches for neurodegenerative biomarker discovery and potential NfL-based multimodal modeling.

AI Method	Algorithm	Input Data	Major Applications	Advantages	Limitations
Supervised Learning	Random Forest	Omics + NfL	Classification	Robust, interpretable	Moderate scalability
Supervised Learning	XGBoost	Multi-omics	Prediction	High accuracy	Hyperparameter tuning
Supervised Learning	SVM	Biomarkers	Disease classification	Good for small datasets	Kernel selection
Unsupervised Learning	K-means	Omics	Patient stratification	Discovers subtypes	Cluster dependence
Unsupervised Learning	Hierarchical Clustering	Omics	Molecular grouping	Easy interpretation	Computationally intensive
Deep Learning	CNN	MRI, PET	Image classification	High performance	Large datasets required
Deep Learning	RNN	Longitudinal biomarkers	Progression prediction	Temporal modeling	Complex training
Deep Learning	Transformers	Multi-modal data	Integrated prediction	Multimodal learning	High computational cost
Explainable AI	SHAP	AI Models	Feature importance	Transparency	Computational burden
Explainable AI	LIME	AI Models	Local interpretation	Clinically interpretable	Local approximation

**Table 6:** AI-based multi-modal data fusion strategies.

Fusion Strategy	Integration Level	Advantages	Limitations	Representative Applications
Early Fusion	Feature level	Captures cross-modal interactions	High dimensionality	Disease classification
Intermediate Fusion	Latent representation	Preserves modality-specific information	Complex architecture	Biomarker discovery
Late Fusion	Decision level	Flexible, interpretable	Limited interaction among features	Multicenter prediction
Hybrid Fusion	Multiple levels	Highest predictive performance	Computationally intensive	Precision neurology

**Table 7:** Current evidence and potential applications of AI-integrated NfL and multi-omics biomarkers.

Clinical Application	Role of NfL	Contribution of Multi-Omics	Role of AI	Current Evidence/Potential Application
Early diagnosis	Detects axonal injury	Disease-specific signatures	Pattern recognition	Emerging, requires external validation
Differential diagnosis	Measures neurodegeneration	Disease discrimination	Classification models	Promising research application
Disease monitoring	Longitudinal biomarker	Molecular progression	Time-series analysis	Relatively established for NfL in selected diseases; AI integration remains investigational
Prognosis	Predicts progression	Risk biomarkers	Outcome prediction	Strong NfL evidence in selected diseases; multimodal AI models emerging
Patient classification	Biological severity	Molecular subtypes	Clustering	Emerging/investigational
Treatment response	Pharmacodynamic marker	Therapeutic targets	Response prediction	Established pharmacodynamic role of NfL in selected settings; AI prediction remains investigational
Clinical trial enrichment	Patient selection	Molecular profiling	Predictive modeling	Promising but requires prospective validation

**Table 8:** Current challenges, limitations and future directions of AI-integrated NFL-based precision neurology.

Challenge	Impact	Current Limitation	Potential Solution	Future Outlook
Biological variability	Reduced specificity	Age, comorbidities	Multimodal biomarkers	Personalized reference ranges
Data standardization	Poor reproducibility	Platform variability	Standardized protocols	International harmonization
Multi-center reproducibility	Limited generalizability	Cohort differences	Federated learning	Global validation
Small sample size	Overfitting	Rare disease cohorts	International biobanks	Large collaborative datasets
Data privacy and ethics	Limited data sharing	Confidentiality concerns	Privacy-preserving AI	Secure collaboration
Regulatory challenges	Slow clinical translation	Lack of guidelines	Standardized validation	Regulatory frameworks
Future technologies	Limited implementation	Emerging methodologies	Single-cell omics, digital twins	Precision neurology

## Data Availability

No new data were created or analyzed in this study. Data sharing is not applicable to this article.
